# The mammalian Ku70 C-terminus SAP domain is required to repair DNA damage

**DOI:** 10.1093/nar/gkaf499

**Published:** 2025-06-11

**Authors:** Yuan Wang, Michael S Czap, Hailey Kim, Paul M Masaka, Hongxin Wang, Md Amjad Beg, Huimei Lu, Jingmei Liu, Yoke-Chen Chang, Peter J Romanienko, Cristina Montagna, Zhiyuan Shen

**Affiliations:** Department of Radiation Oncology, Rutgers Cancer Institute and Robert Wood Johnson Medical School, Rutgers University, New Brunswick, NJ 08901, United States; Department of Radiation Oncology, Rutgers Cancer Institute and Robert Wood Johnson Medical School, Rutgers University, New Brunswick, NJ 08901, United States; Department of Radiation Oncology, Rutgers Cancer Institute and Robert Wood Johnson Medical School, Rutgers University, New Brunswick, NJ 08901, United States; Department of Radiation Oncology, Rutgers Cancer Institute and Robert Wood Johnson Medical School, Rutgers University, New Brunswick, NJ 08901, United States; Department of Radiation Oncology, Rutgers Cancer Institute and Robert Wood Johnson Medical School, Rutgers University, New Brunswick, NJ 08901, United States; Department of Radiation Oncology, Rutgers Cancer Institute and Robert Wood Johnson Medical School, Rutgers University, New Brunswick, NJ 08901, United States; Department of Radiation Oncology, Rutgers Cancer Institute and Robert Wood Johnson Medical School, Rutgers University, New Brunswick, NJ 08901, United States; Department of Radiation Oncology, Rutgers Cancer Institute and Robert Wood Johnson Medical School, Rutgers University, New Brunswick, NJ 08901, United States; Department of Radiation Oncology, Rutgers Cancer Institute and Robert Wood Johnson Medical School, Rutgers University, New Brunswick, NJ 08901, United States; Genome Editing Shared Resource, Rutgers Cancer Institute and Robert Wood Johnson Medical School, Rutgers University, New Brunswick, NJ 08901, United States; Department of Radiation Oncology, Rutgers Cancer Institute and Robert Wood Johnson Medical School, Rutgers University, New Brunswick, NJ 08901, United States; Department of Radiation Oncology, Rutgers Cancer Institute and Robert Wood Johnson Medical School, Rutgers University, New Brunswick, NJ 08901, United States

## Abstract

The mammalian non-homologous end joining (NHEJ) is required for class switch and V(D)J recombination as well as repairing DNA double-strand breaks (DSBs). Initiated by the binding of Ku70/Ku80 (Ku) dimer to DNA ends and the recruitment of the DNA-dependent protein kinase catalytic subunit, NHEJ plays a key role in DSB repair. While the overall function of Ku70 in NHEJ is well documented, the specific role of its highly conserved C-terminal SAP (SAF-A/B, Acinus, and PIAS) domain remains elusive. In this study, we developed a novel mouse model by deleting the SAP domain but preserving Ku70 nuclear localization and its dimerization ability with Ku80. We found that *Ku70 SAP* deletion (*ΔSAP*) had little effect on class switch and V(D)J recombination or animal development but sensitized the animals and cells to radiation and chemotherapy agents. Ku70-ΔSAP cells exhibited reduced Ku70 recruitment and dampened DNA ligase IV retention to DNA damage sites after radiation exposure and displayed a spreading pattern of DSB marker γH2AX after DNA damage. Our findings suggest that the SAP domain is required for cells to optimally cope with DNA damage, making it a potential target to modulate cell sensitivity to therapeutic DSB-inducing agents without interfering with the developmental function of Ku70.

## Graphical Abstract

**Graphical Abstract:**
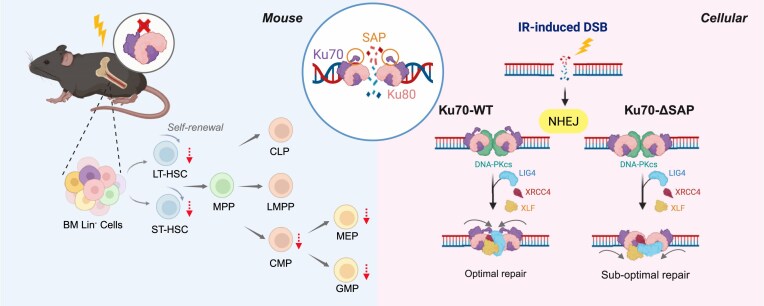

## Introduction

Double-strand breaks (DSBs) are the most lethal form of DNA damage, which can be induced by both endogenous and exogenous factors [1, 2]. The endogenous DSBs can be produced by enzymatic actions during normal DNA processing such as replication and recombination, as well as by free radicals and genotoxic intermediates generated during cellular metabolism. The exogenous DSBs are produced by exposure to environmental agents such as ionizing radiation (IR) and radiomimetic chemotherapy agents. Efficient and precise DSB repair is critical for cell survival and the stability of their genomes. DSBs are mainly repaired by the non-homologous end joining (NHEJ) and homologous recombinational repair (HRR) machineries. In mammalian cells, NHEJ defects have more profound sensitivity to IR than HRR defects [3, 4], reflecting the prominent role of NHEJ in repairing radiation-induced DSBs. It has long been known that the end loading and encircling of the double-stranded DNA (dsDNA) through the aperture-like structure formed between the Ku dimer are the initiating events for NHEJ [5]. However, it is only in recent years that several structural analyses have collectively painted an elegant picture of how a Ku-initiated large protein complex evolves to align two DSB ends for rejoining [6–10]. The DNA-bound Ku dimers recruit DNA-dependent protein kinase catalytic subunit (DNA-PKcs) and then slide inward for about 15 nt to allow DNA-PKcs to occupy the DNA ends [11]. Recruitments of additional proteins including DNA ligase IV (LIG4), X-ray repair cross-complementing 4 (XRCC4), and XRCC4-like factor (XLF) form a long-range synapsis protein complex to hold two DNA ends along with two DNA-PK complexes in proximity [12]. After the synapsed DNA ends are processed and become ligation-compatible, autophosphorylation of DNA-PKcs results in its eviction to transform the long-range synapsis complex to a short-range one that brings the two DSB ends to a close proximity for ligation by an iterative mechanism [8]. Later, a presumable LIG4 reconfiguration allows its ligase domain to reach the dsDNA ends and ligate, one strand at a time by each of the two LIG4 molecules in the short-range synapsis complex [6–8].

In human, the Ku70 subunit is a protein with 609 amino acids (aa). The N-terminal 538 aa are responsible for forming a stable heterodimer with Ku80 and the C-terminal 71 aa consist of a flexible linker region (aa 539–558) that contains the nuclear localization signal (NLS), and a globular domain of 51 aa (aa 559–609) that was historically referred to as the Ku70-SAP (SAF-A/B, Acinus, and PIAS) domain [13]. It is noteworthy that only the C-terminal 37 aa (aa 573–609) are originally defined as the canonical SAP (cSAP) motif [14], while the 14 aa helical domain (referred to as nSAP, aa 559–572) is unique to Ku70 (Supplementary Fig. S1). In some of the crystallography and Cryogenic Electron Microscopy (CryoEM) studies [8, 12], the C-terminal linker and Ku70-SAP were not visualized in the DNA-PK complex, likely due to the flexibility of the linker region and mobility of the Ku70-SAP domain. Others were able to capture at least three different Ku70-SAP positions in the DNA-bound and DNA-free forms of the Ku dimer [9, 10, 15]. Initially, the Ku70-SAP was shown to associate with the α/β-domain of Ku80 of the DNA-free Ku dimer [9, 10]. Using a full-length Ku70 and C-terminally truncated Ku80 (trKu80), it was recently shown that the Ku70-SAP domain can also locate near the aperture of the DNA-free Ku dimer, but may move away from the aperture to an area near aa 450–538 of Ku70 after the Ku70/trKu80 dimer binds to DNA [15]. These works suggested that the Ku70-SAP may change positions in a dynamic NHEJ complex depending on the stage of the repair process, indicating a regulatory or coordinating role of the Ku70-SAP domain. Interestingly, in all these scenarios, it was the nSAP helical domain that was involved in interacting with the main body of the Ku dimer at various positions [9, 10, 15].

Given its ability to bind RNA and DNA [16–18] and the conservative feature of the SAP sequence, there is substantial conjecture surrounding the essential role of SAP domain in Ku70-associated processes. However, experimental evidence regarding its specific role in Ku70 function remains inconclusive, as structural analyses have yet to offer a definitive confirmation or refutation. Engineered deletion of the plant *Arabidopsis thaliana* at Ku70-SAP (aa 593–621), corresponding to human aa 581–609 of the cSAP, resulted in decreased Ku loading to DNA ends *in vitro*. However, this alteration did not have an impact on classical NHEJ, telomere maintenance, or plant development [17].

To directly investigate the biological consequence of mammalian SAP deletion, we used CRISPR technique to generate a novel transgenic mouse model in which the Ku70-SAP domain (aa 564–608) was deleted while the flexible linker with NLS was retained. We found that deletion of the Ku70-SAP had little impact on the stability of Ku80 protein, animal growth, the class-switch recombination (CSR), and V(D)J recombination. However, it did sensitize animals to IR-induced bone marrow (BM) failure, elevated chromosome breakages and aberrations, heightened cell sensitivity to radiation and DSB-inducing chemotherapy agents, reduced Ku70 recruitment to DNA damage sites, and dampened LIG4 and XLF association with chromatin. Our findings suggest a critical role of the SAP domain in repairing DNA damage caused by radiation and chemotherapy agents. Our study represents a pioneering investigation confirming the significant biological role of the Ku70-SAP domain in the repair of DNA damage, mediated through a regulation of the LIG4 activity at the DSB sites. Moreover, it further indicates that targeting the SAP-related function(s) of Ku70 could be a viable strategy to sensitize mammalian cells to therapeutic DNA damage with minimum impact on the developmental function of Ku70.

## Materials and methods

### Mouse line and total body irradiation

**Figure 1.**
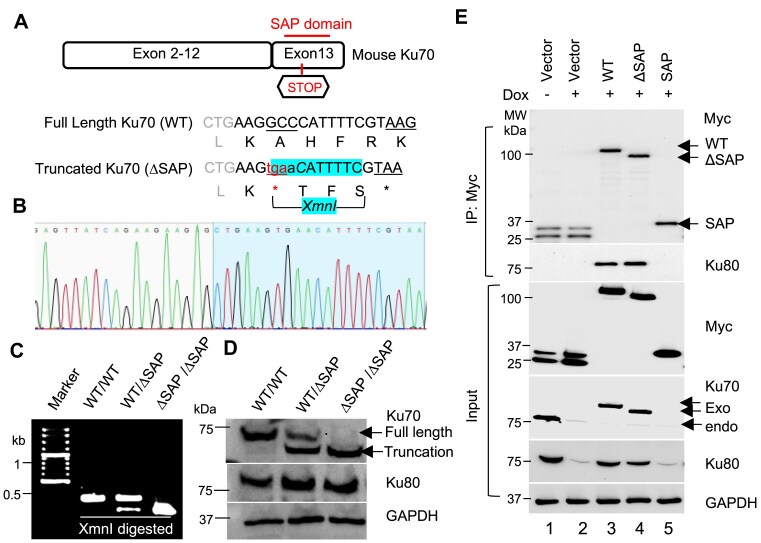
Generation and verification of *Ku70-ΔSAP* mouse line. (**A**) Scheme of the CRISPR–Cas9 editing strategy for mouse *Ku70* gene. The upper sequence indicates the WT mouse Ku70 nucleotide and amino acid sequences starting from L562, and the lower sequence indicates A564X mouse line (ΔSAP) in which GCCC was changed to tGAAC, resulting in one nucleotide insertion and the creation of a new STOP codon to terminate the mouse Ku70 at A564. This also generates a new XmnI (GAANN^NNTTC) site highlighted to be used for genotyping. (**B**) ABI sequencing chromatogram confirming the insertion of a STOP codon. DNA from homozygous *Ku70-ΔSAP* mouse was used for ABI Sanger sequencing. (**C**) Agarose gel electrophoresis of PCR (polymerase chain reaction)-amplified products with XmnI digestion from *WT* and *ΔSAP* mice. (**D**) The expression of full-length and truncated Ku70 in mouse embryonic fibroblasts (MEFs) established from the indicated *Ku70* KI mouse lines. KU80 was detected as the binding partner of Ku70. GAPDH was used as the loading control. (**E**) Co-precipitation of endogenous KU80 with exogenously expressed Ku70 variants. Variants of Myc-EGFP-tagged Ku70 were stably expressed in H1299-shKu70 cells. Cells were treated with or without (doxycycline) Dox for 7 days to induce the knockdown of endogenous Ku70, and the cell extracts were precipitated with anti-Myc conjugated beads. The precipitates were analyzed with western blotting to detect co-precipitated endogenous Ku80.

A Ku70-A564X knock-in (KI) mouse line (ΔSAP) was generated using a single guide RNA (gRNA) and a mixture of two templates adjacent to the Cas9 cutting site. Briefly, the wild-type (WT) mouse Ku70 (mKu70) nucleotide and amino acid sequences starting from A564 were modified by changing GCCC to tGAAC. This resulted in the insertion of one nucleotide and the creation of a stop codon, terminating mKu70 at A564. Additionally, this introduced a new XmnI (GAANN^NNTTC) restriction site, which is highlighted in blue in Fig. 1A and used for genotyping purpose. Mice were housed in individually ventilated cages within a specific pathogen-free facility, maintained on a 12-h light/dark cycle, and provided with *ad libitum* access to food and water. All animal works presented in this study were approved by the Institutional Animal Care and Use Committee at Rutgers Robert Johnson Medical School. We adhered to institutional guidelines regarding animal welfare. For total body irradiation (TBI) experiments, gender-matched mice were temporarily restrained in the ventilated pie-shaped chambers of the RadDisk cage. This cage is circular with eight pie-shaped chambers and a rotating lid that covers seven of the chambers. Up to seven mice were individually placed in the separate chambers of the RadDisk cage each time, which was then placed inside the Gammacell 40 Extractor (MDS Nordion) ^137^Cs γ-irradiator, at a current dose rate of 0.76 Gy/min. Specific radiation doses are provided in the relevant experimental descriptions.

### PCR genotyping and XmnI digestion

The genomic DNA (gDNA) was extracted from 1–2 mm tail tissue and incubated with a lysis buffer [25 mM NaOH, 0.2 mM ethylenediaminetetraacetic acid (EDTA)] in a PCR machine for 30 min at 95°C, followed by cooling to room temperature (RT). An equal volume of neutralization solution (40 mM Tris–HCl, pH 5.0) was then added, and the sample was mixed by shaking several times. For the PCR reactions, 1 μl of the gDNA was used in 10 μl reactions with mKu70 PCR primers: 5′-TGTAGCTGGGCTATCACTGGAAGC-3′ (forward) and 5′-CTTTGTAGCTACCACCTAGCTTGG-3′ (reverse), which flank a region containing a newly introduced XmnI digestion site. PCR amplification was performed using a PCR premix solution (Syd Labs, MB067-EQ2B-L). Following PCR, the product was digested with the XmnI enzyme (NEB, R0194L) at 37°C for 3 h. The digested PCR product was then loaded for agarose gel electrophoresis.

### Collection of lineage-negative cells and flow cytometric analysis

To perform hematopoietic lineage analysis, single-cell suspensions of the BM were prepared from either one nonirradiated mouse or two irradiated mice using their tibia and femur pair and the spine. Following bone crush in RPMI 1640 medium supplemented with 10% fetal bovine serum (FBS), 1% glutamine, 1% penicillin/streptomycin, and 0.01% 2-mercaptoethanol, red blood cells (RBCs) were lysed using RBC lysis buffer (Sigma–Aldrich, R7757). The lineage cell depletion kit (Miltenyi Biotec, #130-090-858) was used to enrich the lineage-negative (Lin^−^) cells. Briefly, the cell pellet was resuspended in FACS buffer [phosphate buffered saline (PBS) with 1% bovine serum albumin (BSA)] containing 20 μl of biotin-antibody cocktail for 10 min, followed by the addition of 40 μl of anti-biotin microbeads for another 15 min on ice. After incubation, cells were washed once with FACS buffer and then loaded onto a pre-wet LS column (Miltenyi Biotec, #130-042-401) on the MACS Multistand (Miltenyi Biotec, #130-108-936) using the magnetic MidiMACS separator (Miltenyi Biotec, #130-042-302). The Lin^−^ cells were collected from the effluent. For flow cytometric analysis, cells were stained using Fixable Yellow Live/Dead Dye (eBioscience, #65-0866-14) and specific antibodies as described [19]. Following staining, cells were washed twice with FACS buffer and prepared for flow cytometry (Cytek Aurora Analyzer, SKU IMSR-4.6a). FlowJo™ portal (v10.9.0) was used for the analysis. UltraComp ebeads™ compensation beads (Invitrogen, #01-2222-42) were used for compensation setup.

### Isolation and activation of mouse splenic B cells

The mouse spleen was placed into a 60-mm petri dish containing B-cell wash buffer (1× Hanks with 1% heat-inactivated FBS and 1% penicillin–streptomycin) and minced into small pieces using a razor blade. The tissues along with the buffer were then transferred into a 70-μm strainer within a 50-ml tube. The spleen fragments were further gently disrupted within the mesh using the flat end of a syringe, and the cells were collected from the 50-ml tube following centrifugation at 1200 rpm for 10 min. The resultant cell pellet was treated with ACK lysing buffer (Gibco, #A1049201) and neutralized by adding wash buffer, followed by another round of centrifugation. The cell pellet was gently tapped to loosen it and then resuspended in 1 ml of wash buffer containing 50 μl of CD43 (Ly-48) MACS microbeads (Miltenyi Biotec, #130-049-801). The mixture was incubated on ice for 30 min. After incubation, the cells were washed by adding wash buffer, gently mixing, and centrifuging at 1200 rpm for 10 min. The sample was resuspended in 1 ml of wash buffer and B cells were separated from the effluent fraction using a MidiMACS™ Separator with an LS column, as described in the Lin^−^ cell collection section. The column was washed twice, and the appropriate cells were placed in a six-well plate or flask containing B-cell culture media (RPMI 1640, 10% heat-inactivated FBS, 1% penicillin–streptomycin, 1% glutamine, 1× nonessential amino acids, 1% sodium pyruvate, 53 mM 2-mercaptoethanol, and 10 mM HEPES) supplemented with 25 μg/ml LPS (Sigma, L2630) and 5 ng/ml IL-4 (Sigma, I1020). The cells were cultured for 72 h at 37°C in a 5% CO_2_ incubator.

### Analysis of metaphase chromosomes

The isolated primary mouse splenic B cells were activated with 7.5 μg/ml concanavalin A (Sigma, C5275) and 25 μg/ml LPS (Sigma, L2630) for 72 h, and then irradiated with 2 Gy of γ-rays using the Gammacell 40 Extractor (MDS Nordion). Following a 12–16 h of incubation post-irradiation, the cells were cultured in the presence of 10 μg/ml Colcemid solution (Roche, #295-892) for 30 min to enrich for metaphase cells. The metaphase cells were collected, swollen in pre-warmed 0.075 M KCl at 37°C for 25 min, and then fixed in a freshly prepared 3:1 mixture of methanol and acetic acid. Cell preparations were dropped onto slides and allowed to dry overnight. The slides were then stained using a DAPI Fluoromount G medium (SouthernBiotech, #0100-20) for visualization. Metaphase images were acquired with a BioView scanning system mounted on an Olympus BX63 microscope (BioView USA Inc., Billerica, MA) with fine focusing oil immersion lens (×60, NA 1.35 oil). Multiple focal planes (*n* = 11, 0.5 μm per plane) were acquired to ensure that scanning on different focal planes was included. Because the frequency of chromosome aberration does not fit with a Gaussian distribution but best with a Poisson distribution, we used Poisson log-linear model to analyze aberration distribution and frequencies. We calculated the rates for each experimental condition and reported the rate ratios along with their 95% confidence intervals.

### Measurements of CSR and V(D)J recombination

The isolated B cells were activated for 72 h, washed with cold PBS, blocked with anti-CD16/32 antibody (BD Biosciences, #553141) at RT for 10 min, and stained with FITC anti-B220 (BD Biosciences, #553088), PE anti-IgM (BD Biosciences, #562033), and Biotin anti-IgG1 (BD Biosciences, #553441) antibodies at 4°C for 30 min. Following staining, the cells were centrifuged and washed twice with PBS, and then stained with Streptavidin Alexa Fluor™ 647 conjugate antibody (Invitrogen, S32357) for an additional 10 min at RT. Flow cytometry was then used for analysis. For the measurement of V(D)J recombination, proteinase K-treated, and phenol-extracted gDNA was isolated from BM cells of 6–8-week-old mice. NSG (NOD scid gamma) mouse was used for comparison. To detect the V(D)J recombination products at the specified genomic locus, primers specific to immunoglobulin D-J_H_ and V-DJ_H_ rearrangements, as described by Ouyang *et al.* [20], were used.

### Micro-irradiation and immunofluorescence staining

MEFs were seeded in chamber slides with 1 μM IdU 1 day prior to micro-irradiation. Laser micro-irradiation was performed using a PALM MicroBeam Laser-capture Microdissection System (Germany) equipped with a Zeiss Axio observer microscope (Carl Zeiss AG) on a custom-designed granite plate. A LabTek chamber slide with live cells was mounted on the microscope stage integrated with the PALM Microlaser workstation. The cells were visualized under visible light, laser-targeted nuclei were selected using a Zeiss software (PALM Robo V4.9), and the nuclei were subsequently irradiated with a pulsed solid-state UV-A laser coupled to the bright-field path of the microscope focused through an LD 40× objective lens. The laser settings were optimized to generate DNA damage restricted to the laser path, dependent on pre-sensitization, with minimal cellular toxicity. At the indicated time points after micro-irradiation, cells were fixed with Formalde-Fresh Solution (Fisher, SF93-4) for 15 min and then permeabilized with 0.15% Triton X-100 for 15 min. After three washes with PBS, the fixed cells were incubated with primary antibodies in 2% BSA blocking buffer at RT for 1 h, followed by incubation with FITC or TRITC conjugated anti-mouse or anti-rabbit secondary antibodies at RT for 1 h. Nuclei were visualized by DNA staining with DAPI Fluoromount G medium. For Ku70 staining, cells were incubated for 5 min at RT with CSK buffer (10 mM Pipes, pH 7.0, 100 mM NaCl, 300 mM sucrose, and 3 mM MgCl_2_) containing 0.1% Triton X-100 and 0.3 mg/ml RNase A before the fixation step [21].

### Doxycycline-inducible Ku70 knockdown in human H1299 cells

To complement Ku70 deficiency in human cells with variants of Ku70, we used a previously developed Dox-inducible Ku70 knockdown system, now termed H1299-ishKu70 cell line, which contains a Dox-inducible short hairpin RNA (shRNA) expression cassette that target human Ku70 messenger RNA [22]. Briefly, Ku70 shRNA targeting the 3′-UTR of the *hKu70* gene was cloned into the pLKO-Tet-Neo vector, which confers neomycin resistance. For lentivirus production, 293T cells were co-transfected with pLKO-shKu70-Neo, psPAX2, and pMD2G at a 2:1:1 ratio. Seventy-two hours post-transfection, the virus-containing supernatant was collected and filtered through a 0.45-μm nylon mesh and used to infect H1299 cells in the presence of 8 μg/ml polybrene. The infection process was repeated twice. Following infection, the cells were allowed to recover overnight in fresh medium and were then subjected to selection with 800 μg/ml neomycin for 7 days. The WT and truncated complementary DNA of hKu70 variants were cloned into the pLXSP-Myc-EGFP vector, which provides puromycin resistance. The DNA constructs were transfected into Phenix A cells to generate retrovirus. H1299-ishKu70 cells were then infected with the produced retrovirus through three infection cycles (8 h of infection followed by 16 h of incubation with fresh medium per cycle). Post-infection, the cells were subjected to selection with 1 μg/ml puromycin. Stable cell lines were established by isolating and expanding positive single clones. The cells were cultured in α-minimum essential medium (α-MEM) supplemented with 10% FBS, 20 mM glutamine, and 1% penicillin–streptomycin at 37°C in a 5% CO_2_ incubator.

### Generation of MEFs from Ku70-ΔSAP mice

*Ku70-ΔSAP* heterozygous mice were bred at 3 months of age, and upon confirmation of pregnancy, female was euthanized to harvest E15.5 embryos. The MEFs were generated as previously described [22]. Briefly, embryos were extracted from the uterus. The skin from the back of each embryo was removed, minced, and subjected to enzymatic digestion in 0.25% trypsin–EDTA solution at 37°C for 30 min. Following digestion, α-MEM with 10% FBS was added to inactivate the trypsin. The mixture was gently agitated, and the cell suspension was left undisturbed for about 5 min to allow larger embryo fragments to settle. The supernatant containing single cells was then transferred to a new tube and centrifuged at 1000 rpm for 3 min. The resulting cell pellet was resuspended in α-MEM supplemented with 10% FBS, 20 mM glutamine, and 1% penicillin–streptomycin, and the cells were cultured at 37°C in a 5% CO_2_ incubator. The MEFs were initially prepared and then passaged repeatedly until passage 30 for aliquots and storage. These MEFs maintained stable growth rates at least by passage 55 in the same α-MEM with 10% FBS. Cells between passages 30 and 35 were used for radiation sensitivity and DNA damage assays in this study.

### Colony formation assay after radiation exposure, and measurement of cell sensitivity to chemotherapy agents

MEFs were seeded in 100-mm petri dishes at a density of 2500 cells per dish in triplicates, and allowed to adhere overnight prior to exposure to IR. The MEFs were cultured in α-MEM supplemented with 20% FBS to facilitate colony formation. Human H1299-ishKu70 cells expressing Ku70 variants were pre-treated with Dox for 7 days to knock down endogenous Ku70 expression before the colony formation assay was performed. Following the Dox treatment, H1299-ishKu70 cells were seeded in 100-mm petri dishes at varying densities according to the radiation dose: 200 cells for the control group (0 Gy), 1000 cells for the 2 Gy group, 5000 cells for the 4 Gy group, and 25 000 cells for the 6 Gy group, with each condition performed in triplicates. The cells were cultured in α-MEM supplemented with 10% FBS and in the presence of Dox. Fourteen days after IR exposure, the cells were fixed using a 0.5% crystal violet solution prepared in 20% methanol for subsequent staining and colony counting. To measure cell sensitivity to chemotherapy drug, cells were plated into multi-well plates and treated with varying concentration of drugs as specified in the “Results” section and figures. The cell growth and viability were measured every 2 h using the IncuCyte live-cell imaging system.

### Antibodies

The following primary antibodies were used: Ku70 (Invitrogen, MA5-42413; Santa Cruz, sc-56129), Ku80 (Santa Cruz, sc-5280), GAPDH (Santa Cruz, sc-365062), γH2AX (Santa Cruz, sc-517348; Cell Signaling Technology, #2577L), Myc (Santa Cruz, sc-40); XRCC4 (Santa Cruz, sc-365118), XLF (Santa Cruz, sc-393844), LIG4 (Santa Cruz, sc-28232; Cell Signaling Technology, #14649S), total DNA-PKcs (Cell Signaling Technology, #12311S), phosphorylated DNA-PKcs (S2056) (Abcam, ab-18192-100), histone H4 (Cell Signaling Technology, #2935T), and histone H3 (Cell Signaling Technology, #9715).

### Protein fractionation analyses

H1299-ishKu70 cell lines stably expressing Myc-EGFP-tagged Ku70 (WT and mutants) were treated with 0.2 μg/ml Dox for 7 days to knock down endogenous Ku70, and then treated with or without γ-rays at 6.5 Gy at the current dose rate of 0.76 Gy/min. At 30 min post-IR, the cells were collected to separate each protein fraction. Briefly, the cell pellet was incubated on ice with permeabilization buffer (10 mM HEPES, pH 7.4, 10 mM KCl, 0.05% NP-40) supplemented with protease inhibitors to isolate the cytosolic fraction (S1). After a wash with permeabilization buffer, the cell pellet was incubated with nuclease reaction buffer (10 mM HEPES, pH 7.4, 10 mM KCl, 0.5 mM MgCl_2_, 2 mM CaCl2) supplemented with Benzonase nuclease for 30 min at 4°C to obtain the soluble nuclear fraction (S2). The total soluble fraction was created by pooling S1 and S2 fractions in a 1:1 ratio. The remaining nuclear pellets were processed using two approaches. To extract all chromatin-associated proteins, the pellets were dissolved with 0.2 N HCl on ice for 10 min, and the soluble supernatant was then neutralized with an equal volume of 1 M Tris–HCl (pH 8.0) and saved as the whole chromatin-associated fraction (S3W). To further differentiate between loosely and tightly bound chromatin proteins, the nuclease-treated nuclear pellets were treated with IV150 buffer (10 mM Tris, pH 7.4, 2 mM MgCl_2_, 2 mM EGTA, 0.1% Triton X-100, and 140 mM NaCl) for 30 min with rotation at 4°C. The soluble supernatants were collected as the loosely associated chromatin fraction (S3L). The use of 150 mM salt buffer is aimed at extracting loosely bound chromatin proteins such as HMGB1, leaving behind tightly bound proteins [23]. The remaining salt-resistant pellets were then extracted with 0.2 N HCl on ice for 10 min, and the supernatant was neutralized with an equal volume of 1 M Tris–HCl (pH 8.0) to obtain the tightly bound chromatin proteins (S3T).

### Comet assay

The alkaline comet assay was performed to measure cellular DNA damage by using the Comet Assay Kit (Abcam, ab238544). MEFs were either untreated or exposed to 30 Gy of IR and collected at 15 and 120 min after IR exposure. The cell samples were mixed with comet agarose and applied to slides pre-coated with a base layer of the comet agarose. After treatment with lysis buffer and an alkaline solution, electrophoresis was carried out using a cold alkaline electrophoresis solution for 30 min at a voltage of 1 V/cm. The slides were then rinsed with pre-chilled deionized water and fixed in 70% cold ethanol for 5 min. Once air-dried, the slides were stained with Vista Green DNA Dye and analyzed under a fluorescence microscope. Key metrics such as the percentage of DNA in the tail, tail length, and tail moment were quantified using the ImageJ plugin, OpenComet, by evaluating over 35 nuclei from each group.

## Results

### Deletion of mouse Ku70-SAP domain had minimal impact on animal growth, spontaneous tumor formation, class switch of immunoglobulins, and V(D)J recombination in B cells

Despite speculation about the role of the SAP domain in Ku70 functions based on *in vitro* biochemical analyses [16–18, 24, 25], structural analysis has not provided a clear indication of whether the Ku70-SAP domain is essential for the NHEJ complex, and it remains unknown whether Ku70-SAP has a functional role in mammalian development and the repair of genotoxic damage. To directly address this long-standing knowledge gap, we resorted to the CRISPR knock-in approach to modify the coding sequence in exon 13 of mouse *Ku70* as illustrated in Fig. 1A. This resulted in a new transgenic mouse line, designated as *Ku70-ΔSAP*, which codes for a Ku70 protein lacking the C-terminal SAP domain (aa 564–608) in mice, corresponding to aa 566–609 in humans. It is noteworthy to point out that this deletion included both the nSAP and cSAP regions of Ku70 (Supplementary Fig. S1), while preserving the linker region that contains the NLS in the truncated Ku70-ΔSAP (aa 1–563 in mice, corresponding to aa 1–565 in humans). The authenticity of the homozygous founder line was confirmed by Sanger DNA sequencing of the *Ku70* locus (Fig. 1B). Additionally, the new mouse line was validated by XmnI digestion of PCR-amplified DNA covering the modified region of the *Ku70-ΔSAP* line (Fig. 1C). Western blot analyses of proteins extracted from homozygous and heterozygous *Ku70-ΔSAP* mice confirmed the expression of the truncated form of mouse Ku70 protein (Fig. 1D). In *Ku70* null mice, abolition of the Ku dimer was observed previously [26], along with defective immunoglobulin class switching in B cells and impairment in animal growth [26–28]. However, the *Ku70-ΔSAP* mice showed little reduction in Ku80 protein levels (Fig. 1D), indicating that the truncation does not affect Ku80 protein. To determine whether SAP domain deletion impacts Ku70 and Ku80 interaction, we used a previously reported Dox-inducible Ku70 knockdown system [22], termed H1299-ishKu70, to reduce the endogenous Ku70 levels. We then stably transfected the H1299-ishKu70 cells with either a Myc-EGFP vector or various Myc-EGFP-tagged Ku70 variants, including Ku70-WT, Ku70-ΔSAP, and Ku70-SAP as illustrated in Supplementary Fig. S2. After precipitating with anti-Myc antibodies, we found that endogenous Ku80 was equally co-precipitated by both Ku70-WT and Ku70-ΔSAP, but not by the SAP domain of Ku70 or the vector alone (Fig. 1E), suggesting that Ku70-ΔSAP is sufficient to interact with the endogenous Ku80 protein. This result supports the idea that stabilization of the Ku heterodimer relies on the dimer-forming N-terminal region of Ku70 [26], rather than the C-terminal SAP domain. In contrast to the previously reported phenotypes of smaller body size, shorter lifespan, and spontaneous T-lymphoma development in *Ku70* null mice [27, 29], the *Ku70-ΔSAP* mice exhibited normal body weight (Supplementary Fig. S3A), unchanged overall survival within the observed ∼600 days of age, and insignificant change of spontaneous tumor incidence (Supplementary Fig. S3B). The lack of observable defects in development of our *Ku70-ΔSAP* mice is similar to what were observed in a recent *Ku70* truncation model with aa 558–608 deletion [30].

The *Ku70* null mice exhibit defects of V(D)J recombination and CSR in IgM-positive B cells [20, 26]. To determine whether the Ku70-SAP domain influences lymphocyte development and the conversion of IgM to IgG1, we isolated splenic B cells from the *Ku70-ΔSAP* mouse line. After *in vitro* stimulation, we compared the expression levels of IgG1 between *Ku70-WT* and *-ΔSAP* mice. Despite the anticipated role of Ku70-SAP domain in the NHEJ, our results showed that its deletion did not affect the conversion from IgM to IgG1 in mouse splenic B cells, as compared to their sex-matched WT littermates (Supplementary Fig. S3C), indicating a minimum role of Ku70-SAP in the CSR. Using the strategy reported [20], we did observe the comparable V(D)J recombination in the B cells of *Ku70-ΔSAP* and *WT* mice (Supplementary Fig. S3D). In addition, we did not observe any significant changes in lymphocytes or other peripheral blood components in the *Ku70-ΔSAP* mice at 180 days of age (Supplementary Table S1), supporting the notion that the SAP domain is not essential for the development of lymphocytes.

### Deletion of mouse *Ku70-SAP* domain sensitized mice to TBI

The current structural models of the DNA bound DNA-PK complex suggest that an intermediate step is involved in protecting and rendering them ligation-compatible before the transition from long-range synapsis complex to the short-range complex for end ligation [6, 8, 31]. Interestingly, two distinct long-range complexes have been observed, each handling different scenarios of end processing and ligation [8], indicating that at least two types of intermediate steps may have evolved to accommodate different types of DNA ends. This makes biological sense because the processes for processing and religating V(D)J segments or CSR ends would differ from those required to ligate DSB ends generated by IR, which often have nonuniform chemical structures that are not immediately suitable for end extension, ligation, etc. Therefore, it is important to determine whether the SAP deletion affects the repair of radiation-induced DSBs, despite having little impact on CSR, V(D)J recombination, and lymphocyte development. With this understanding, we determined whether the SAP deletion affects Ku70’s ability to manage IR-induced DNA damage at the whole-animal level.

**Figure 2.**
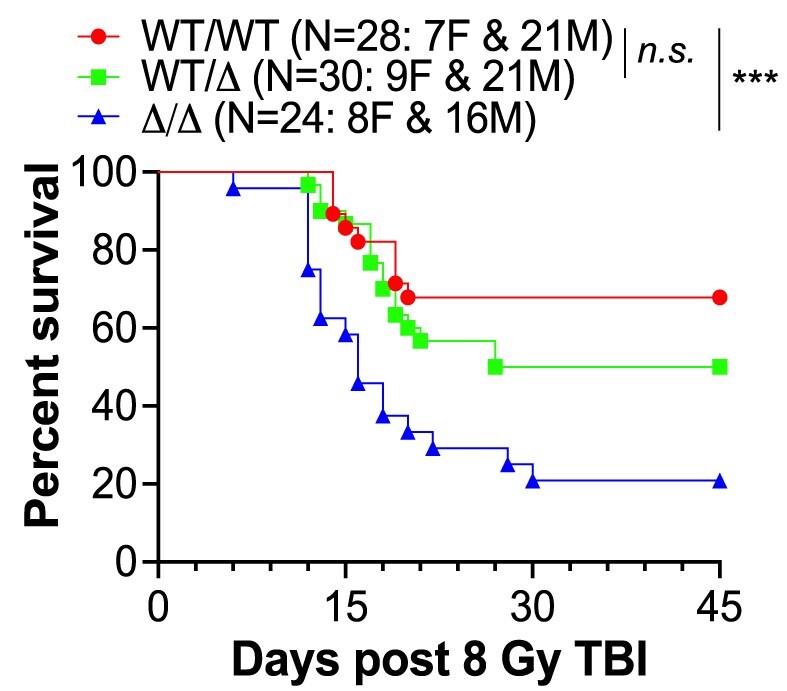
Kaplan–Meier survival curves of *Ku70* KI mice after 8 Gy of TBI. Eight-weeks-old littermates of WT (*WT/WT*), heterozygous *Ku70-ΔSAP* (*WT/Δ*), and homozygous *Ku70-ΔSAP* (*Δ/Δ*) were exposed to a single dose of 8 Gy γ-radiation by TBI. The numbers of male (M) and female (F) mice in each group are shown. Log-rank Mantel–Cox test was used for statistical analysis. ***p ≤ 0.001. n.s. denotes nonsignificant.

It is well established that mammalian hematopoietic stem cells (HSCs) and hematopoietic progenitor cells (HPCs) are among the cell types most sensitive to IR-induced DNA damage. TBI-induced killing of HSCs and HPCs impairs hematopoiesis, which is essential for replenishing the functional pool of blood components. This impairment leads to BM failure and often results in animal death, typically occurring within 1–3 weeks post-TBI. To test whether the SAP deletion confers a sensitization of the BM to radiation damage, we exposed sex-matched littermates to an 8 Gy TBI, a dosage known to cause mouse lethality due to BM failure [32]. We observed that the *Ku70-ΔSAP* mice were hypersensitive to IR. As shown in Fig. 2, ∼70% of the *WT* mice survived the TBI, but only 20% of the *Ku70-ΔSAP* mice did so.

**Figure 3.**
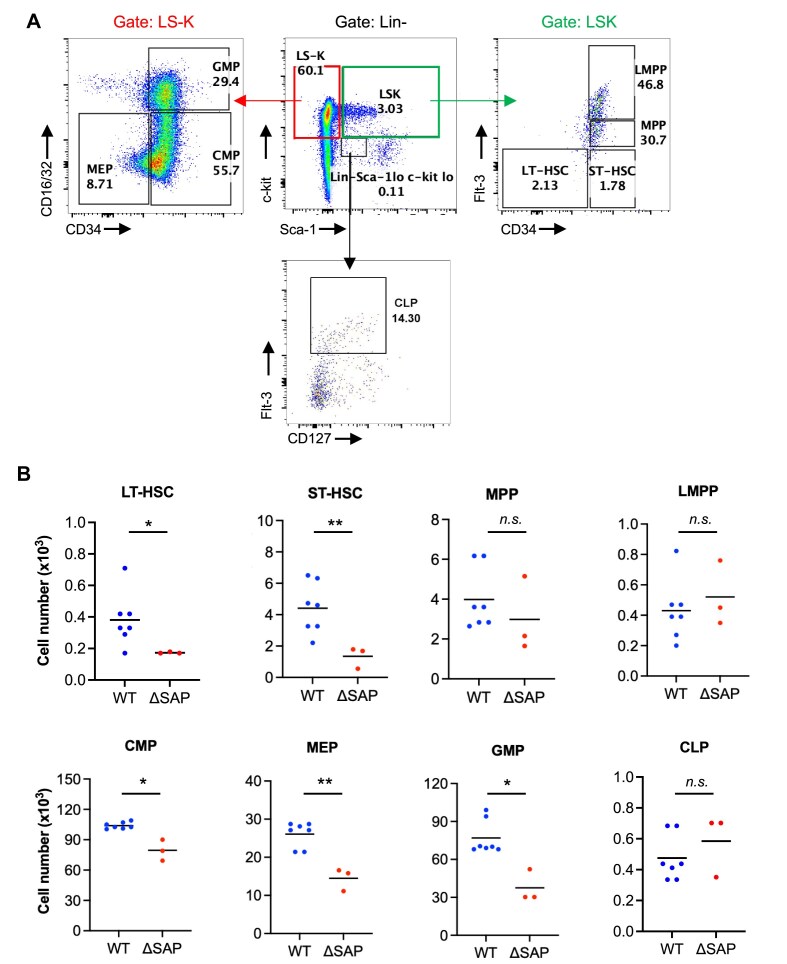
Counts of HSCs and progenitor cells in the BM post-TBI. Eight-weeks-old male mice were exposed to a sub-lethal dose of 6.5 Gy γ-radiation by TBI. The BM cells were collected 14 days post-TBI, and Lin^−^ cells were isolated. (**A**) The flow cytometry gating strategy identified various cell populations: LS-K (Lin^−^, Sca-1^−^, c-kit^+^; left panel) was further divided into CMPs (CD16/32^−^ CD34^+^), GMPs (CD16/32^+^ CD34^+^), and MEPs (CD16/32^−^ CD34^−^). LSK (Lin^−^, Sca-1^+^, c-kit^+^; right panel) was divided into LT-HSCs (CD34^−^ Flt-3^−^), ST-HSCs (CD34^+^ Flt-3^−^), MPPs (CD34^+^ Flt-3^Int^), and LMPPs (CD34^+^ Flt-3^Bright^); Sca-1^low^, c-kit^low^ (bottom panel) was used to gate the CLP population. (**B**) The total numbers of each cell populations are presented as mean (N = 3–5 mice/group). *p ≤ 0.05; **p ≤ 0.01; and *n.s*. denotes nonsignificant by unpaired two-tailed Student’s *t-*test. The BM cells collected from the no-IR groups are shown in Supplementary Fig. S4.

To confirm whether the increased sensitivity of *Ku70-ΔSAP* mice was attributed to the sensitivity of the HSCs to IR, we performed a hematopoietic lineage analysis using a previously established approach [19]. A sublethal radiation dose of 6.5 Gy was administered to ensure the animals remained viable for BM collection and subsequent analysis, which is a standard approach in the field. This assay is purposed to monitor the recovery of the hematopoietic hierarchy after the TBI damage. Briefly, BM mononuclear cells were obtained from the femur, tibia, and spine bones of each mouse. Among the Lin^−^ cells, Sca-1^−^c-Kit^+^ (LS-K) and Sca-1^+^c-Kit^+^ (LSK) cells were further categorized based on cell surface markers using the same approach as reported [19]. Representative flow cytometry profiles are shown for the *Ku70-ΔSAP* mouse (Fig. 3A). In the absence of irradiation, there was no difference in the total number of long-term hematopoietic stem cells (LT-HSCs), short-term hematopoietic stem cells (ST-HSCs), multipotent progenitors (MPPs), lymphoid-primed multipotent progenitors (LMPPs), common myeloid progenitors (CMPs), megakaryocyte-erythroid progenitors (MEPs), granulocyte-monocyte progenitors (GMPs), and common lymphoid progenitors (CLPs) between *Ku70-WT* and *ΔSAP* mice (Supplementary Fig. S4). However, at 14 days post-IR, *Ku70-ΔSAP* mice exhibited a notable decrease in LT-HSCs and ST-HSCs (Fig. 3B). In addition, there were significant reductions in the committed progenitor populations such as CMP, MEP, and GMP (Fig. 3B). The numbers of MPPs, LMPPs, and CLPs did not differ significantly between *Ku70-WT* and *ΔSAP* mice. These findings suggest that the SAP domain of Ku70 plays a crucial role in the survival of hematopoietic stem and certain progenitor populations following IR-induced damage, as well as in the subsequent hematopoiesis required to refurnish the BM and blood.

### SAP domain deletion increased IR-induced chromosome damage

**Figure 4.**
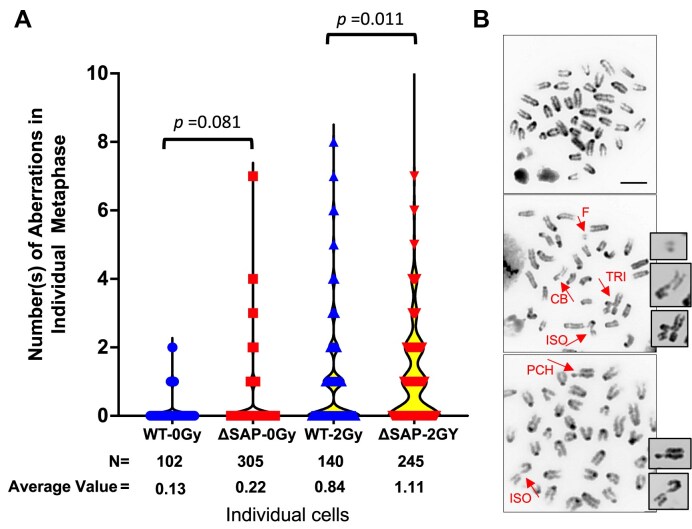
Chromosomal aberrations in *Ku70-WT* and *ΔSAP* mice splenic B cells. The primary splenic B cells were treated with or without 2 Gy IR and then collected for metaphase spread. (**A**) The distribution of number of chromosome aberration(s) in individual metaphase spread. Results were pool from four independent experiments. The numbers of metaphase spreads (N) in each group and the average numbers of aberration in each group are indicated under the *X*-axis. *P*-values were calculated by Poisson linear regression analysis. (**B**) Representative image of DAPI-stained metaphase spreads with aberrations (middle and bottom panels) or without (top panel). Scale bar: 7.7 μm. Abbreviations: CB, chromatid breaks; F, fragment; TRI, triradial; ISO, isochromosome; PCH, premature chromatid separation. See Supplementary Table S2 for all types of aberrations scored.

Aberrations of metaphase chromosomes are a reliable indicator for defective repair of radiation-induced DNA damage. To address whether the SAP deletion impairs the repair of radiation-induced DSBs, we cultured stimulated splenic B lymphocytes, and exposed them to 2 Gy of γ-rays. Then, metaphase spreads were prepared and scored for chromosome aberrations. As shown in Fig. 4, in the absence of radiation exposure, the average chromosome aberration rate for WT cells (WT-0Gy) was 0.13 (95% CI: 0.07, 22), and 0.22 (95% CI: 0.17, 0.28) for Ku70-ΔSAP cells (ΔSAP-0Gy). Despite of the aberration rate ratio of 1:7 (95% CI: 0.94, 3.08) between WT-0Gy and ΔSAP-0Gy, this value is statistically marginal (*p* = 0.081) likely due to the low frequencies of aberration without irradiation. However, following exposure to 2 Gy of γ-rays, the aberration rate was 0.84 (95%CI: 0.70, 1.01) for the WT cells (WT-2Gy) and 1.11 (95% CI: 0.99, 1.25) for Ku70-ΔSAP cells (ΔSAP-2Gy). This 1.32 (95% CI: 1.07, 1.64)-fold of increase in Ku70-ΔSAP cells was statistically significant (*p* = 0.011). As shown in Supplementary Table S2, the increase of aberration was contributed by both chromosome and chromatid breaks, which are known to result from defective DSB repair, including deficiencies in NHEJ throughout the cell cycle.

### SAP domain deletion increased cell sensitivity to DSB-inducing chemotherapy agents and IR

**Figure 5.**
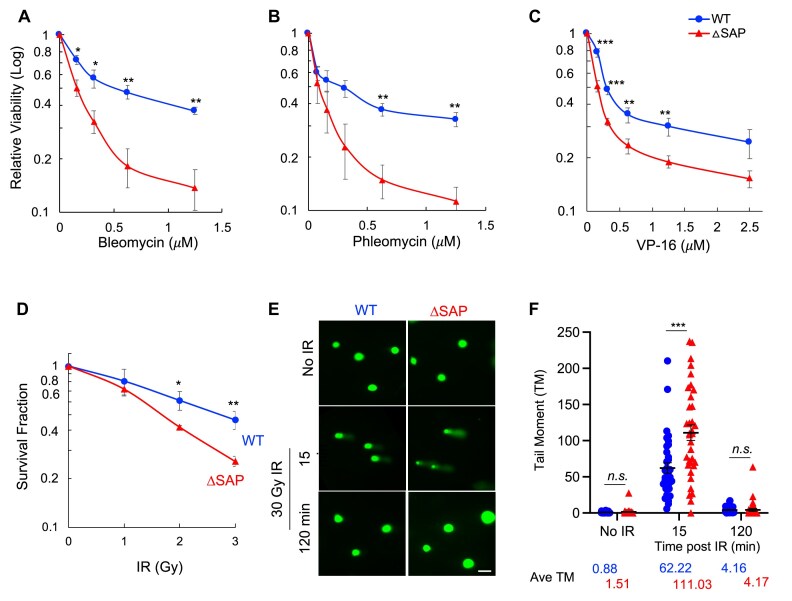
Drug and radiation sensitivities of Ku70-WT and Ku70-ΔSAP cells. iMEFs were established from embryos of littermates derived from a cross between heterozygous Ku70-ΔSAP mice and were used to assess drug sensitivity (A–C) and radiation sensitivity (D–F). The drug sensitivity of iMEFs was evaluated by measuring cell viability every 2 h using the IncuCyte live-cell imager. Relative cell viabilities at 72 h after continuous exposure of (**A**) bleomycin, (**B**) phleomycin, and (**C**) VP16 at the indicated concentrations, as normalized to the same cells without drug treatment. The colony formation assays were performed on iMEFs treated with indicated doses of IR 1 day after seeding (**D**). The colonies were counted 14 days post-irradiation. For the comet assay (E and F), iMEFs were treated with or without IR and collected at different time points. (**E**) Representative comet images; scale bar, 75 μm. (**F**) The tail moment was analyzed using the OpenComet v1.3 software. The average score of the tail moment (Ave TM) is shown at the bottom. Over 35 cells were analyzed from each group. Each experiment was performed at least three times. Error bars are the standard errors of the means (SEMs). *P*-values were calculated based on two-tailed Student’s *t*-test. *p ≤ 0.05; **p ≤ 0.01; ***p ≤ 0.001; *n.s*., nonsignificant.

Having found that mouse *Ku70-SAP* deletion sensitized BM stem cell to IR *in vivo* (Figs 2 and 3) and increased the frequencies of chromosome breaks in cultured B cells (Fig. 4), we employed additional assays to confirm the role of Ku70-SAP in DSB repair. Immortalized mouse embryonic fibroblast (iMEF) lines were established from both *Ku70-WT* and *ΔSAP* mice. These iMEFs were exposed to radiation-mimicking chemotherapy agents bleomycin (Fig. 5A) and phleomycin (Fig. 5B), along with topoisomerase-2 poison VP16 (Fig. 5C) known to induce DSBs. As shown in Fig. 5, the *Ku70-ΔSAP* cells displayed significantly higher sensitivity to these DSB-inducing agents compared to *Ku70-WT* cells. Based on colony formation assay, we found that the *Ku70-ΔSAP* iMEFs were more sensitive to γ-radiation than the *Ku70-WT* cells (Fig. 5D). Next, we subjected the irradiated the iMEFs to alkaline comet assay. As shown in Fig. 5E and F, longer comet tail moments were observed in *Ku70-ΔSAP* cells than in *Ku70-WT* cells shortly after the exposure, but this became insignificant by 2 h after the radiation exposure, indicating a dampened repair in early phase after DNA damage. Furthermore, Ku70-ΔSAP expressed in the Dox-treated H1299-ishKu70 exhibited a radiation sensitization when compared with Ku70-WT that was expressed in the same cell line (Supplementary Fig. S5). Collectively, the results (Figs 2–5 and Supplementary Fig. S5) suggest that deletion of the SAP domain leads to heightened sensitivity to radiation- and chemotherapy drug-induced DNA damage in mice and cells, despite its minimal impact on CSR and V(D)J recombination (Supplementary Fig. S3C and D) and lymphocyte development (Supplementary Table S1).

### Delayed recruitment of Ku70-ΔSAP at DNA damage sites in MEFs

**Figure 6.**
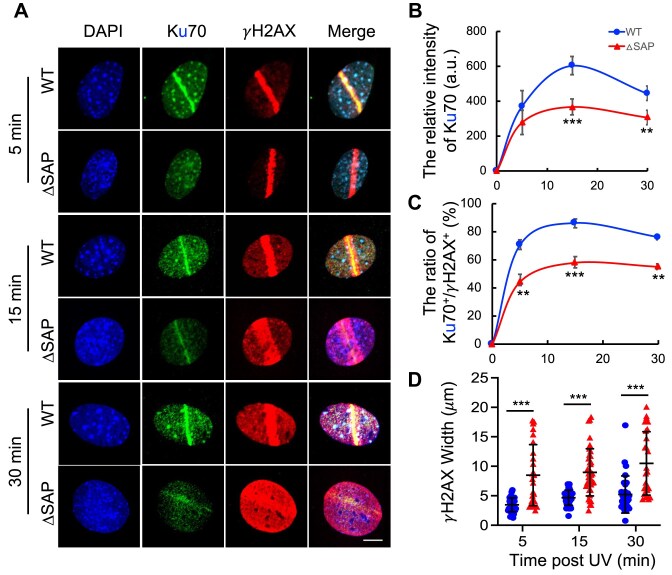
Ku70 recruitment to micro-irradiated DNA damage sites. iMEFs were micro-irradiated and immune-fluorescently stained with appropriate antibodies as described in the “Materials and methods” section. (**A**) Representative confocal images of Ku70 (green) and *γ*H2AX (red) after micro-irradiation at the indicated time points were obtained using the Nikon A1R-Si Confocal Microscope System. Ku70-WT and ΔSAP iMEFs were subjected to micro-irradiation and then treated with a CSK buffer containing RNaseA at intervals of 5, 15, and 30 min following exposure. After treatment, the cells were fixed and subsequently stained with antibodies against γH2AX and Ku70. Cell nuclei were visualized with DAPI (blue). Scale bars, 10 μm. (**B**) The relative fluorescence intensity of Ku70 in WT and ΔSAP iMEFs at the irradiated sites was quantified at the indicated time points after micro-irradiation. (**C**) The ratio of Ku70^+^ and *γ*H2AX^+^ cells. For each time point, >40 nuclei were analyzed in three independent experiments. (**D**) The width of *γ*H2AX in each individual cell was measured using the ImageJ software. Over 35 cells were analyzed from each group. The representative image and the measurement method are shown in Supplementary Fig. S6. *P*-values were calculated using a two-tailed Student’s *t*-test in panels (B) and (D), and binomial *z* statistic pairwise comparison was used for statistical analysis in panel (C). **p ≤ 0.01; ***p ≤ 0.001; *n.s*., nonsignificant.

To explore the cellular mechanisms by which SAP deletion confers a sensitization to DNA damage, we compared the recruitments of Ku70-WT and Ku70-ΔSAP to DNA damage stripes after laser micro-irradiation. By treating cells with CSK buffer supplemented with RNase A [21], we were able to visualize Ku70 recruitment at the micro-irradiated nuclear stripes with minimum background signal in iMEFs (Fig. 6A). Quantifications of Ku70 intensities at the irradiated stripes revealed a significant reduction in Ku70-ΔSAP recruitment compared to WT Ku70 (Fig. 6A and B). Furthermore, a lower percentage of cells with positive recruitment of Ku70-ΔSAP than WT Ku70 as compared with the γH2AX stripe-positive cells was observed (Fig. 6C). For example, 15 min after micro-irradiation, Ku70-ΔSAP showed only 50% of the staining intensity relative to WT Ku70 at the irradiated stripes (Fig. 6B). Additionally, only 50% of the γH2AX-positive cells also exhibited Ku70-ΔSAP positivity, compared to 80% of them positive for Ku70-WT (Fig. 6C). Interestingly, in contrast to the reduced and slimmed Ku70-ΔSAP recruitment and localization at the irradiated stripes, we observed wider stripes and spreading pattern of the γH2AX staining in the Ku70-ΔSAP cells than the WT cells (Fig. 6D and Supplementary Fig. S6). Although the exact implications of this γH2AX pattern remains unclear, it may indicate a persistent DNA damage signaling due to delayed DNA repair or reflect an underlying chromatin structural change that warrants further investigation. Collectively, these findings highlight the essential role of the SAP domain in efficiently recruiting Ku70 to DNA damage sites and repairing DSBs in the live cells.

### Deletion of the SAP domain reduced the retention of LIG4 at the DNA damage sites

**Figure 7.**
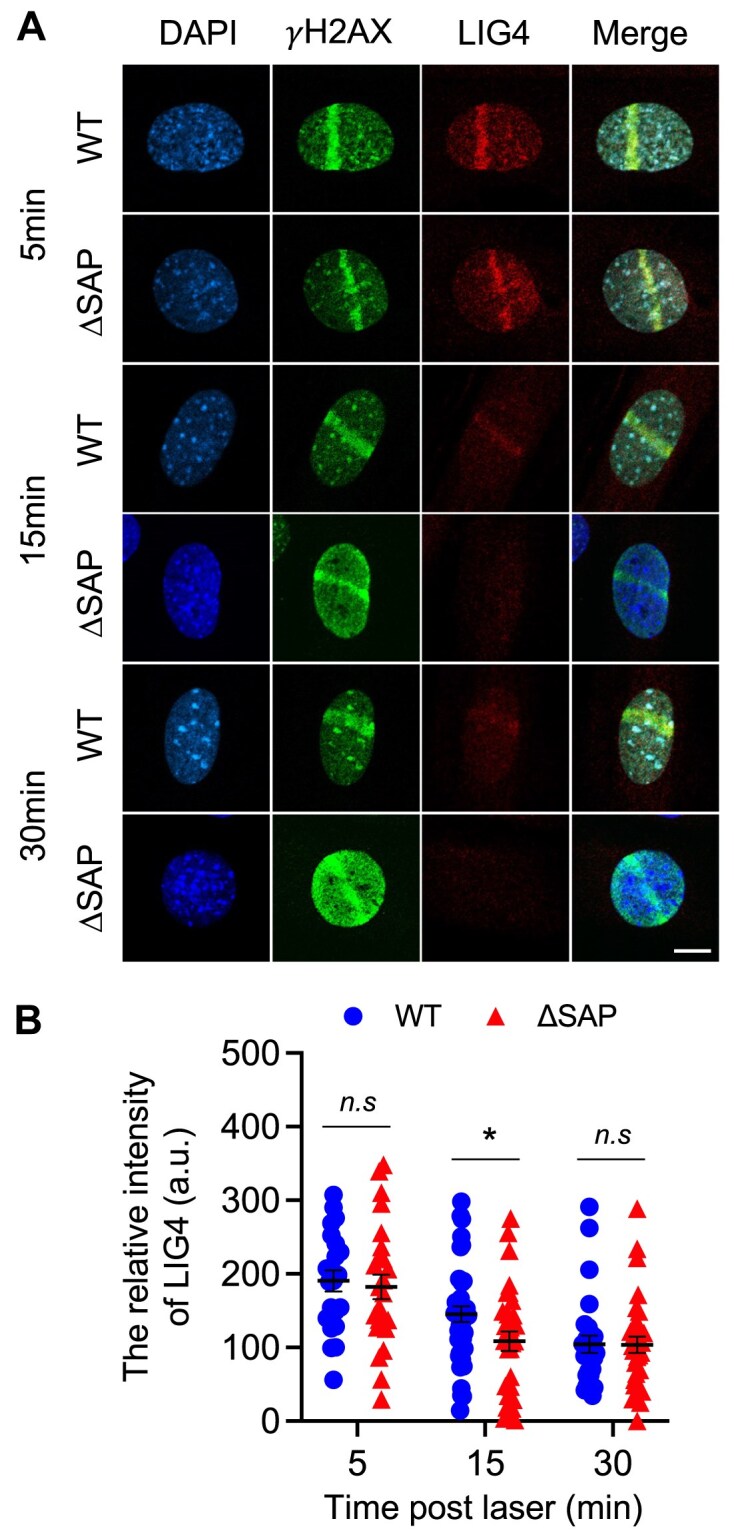
The recruitment of LIG4 to micro-irradiated DNA damage sites. (**A**) Representative images of γH2AX (green) and LIG4 (red) in Ku70-WT or ΔSAP iMEFs after micro-irradiation at the indicated time points. The cells were micro-irradiated and then fixed at intervals of 5, 15, and 30 min following exposure. Then, the cells were stained with antibodies against γH2AX and LIG4. Cell nuclei were visualized with DAPI (blue). Images were obtained from the Nikon A1R-Si Confocal Microscope System. Scale bars, 10 μm. (**B**) The relative fluorescence intensity of LIG4 at the irradiated sites was quantified at the indicated time points after micro-irradiation. For each time point, >20 nuclei were analyzed in three independent experiments. Error bars represent the SEMs. *P*-values were calculated using a two-tailed Student’s *t*-test. *p ≤ 0.05; *n.s*., nonsignificant.

Numerous findings have provided detailed insights into crucial stages of the NHEJ pathway. According to a prevailing model, the DNA-PK complex initially binds to the broken DNA ends, attracting complex of LIG4/XRCC4/XLF. This assembly forms a synaptic complex responsible for tethering and rejoining DNA ends [4, 33–36], which does not need the enzymatic activity of the LIG4 [6, 8, 31]. Given that the SAP deletion affected little of the normal CSR and V(D)J repair process but impacted IR induced DSB repair, our speculation is that the deletion likely interferes with a step occurring after the formation of the initial DNA-PK complex. This step is considered fundamental in a subset of NHEJ events. We also investigated LIG4 as a representative of the LIG4/XRCC4/XLF oligomers. Surprisingly, we noticed a reduced presence of LIG4 at the stripes at an intermediate time point. As shown in Fig. 7, the intensities of recruited LIG4 at 5 min after micro-irradiation were comparable between Ku70-WT and ΔSAP cells. At 15 min, the LIG4 stripe intensities in Ku70-ΔSAP cells dropped significantly more than in WT cells from the 5 min time point. By 30 min, LIG4 intensities in Ku70-WT and Ku70-ΔSAP cells were similar, because LIG4 levels in the WT cells continued to decline while they no longer drop in the Ku70-ΔSAP after the earlier decline before 15 min. This pattern suggests that while the SAP domain may not be essential for initially recruiting LIG4 to damage sites, its deletion results in an earlier decline in LIG4 intensity at the micro-irradiated stripes, suggesting a dampened retention of LIG4 at the damage sites. This finding implies that the SAP domain may play a role in optimal retention of LIG4 at the damage sites, even though it might not be indispensable for the initial recruitment. We also monitored the intensities of 53BP1 at the micro-radiated stripes, which may associate with severity of residual DNA damage. At 15 min post-irradiation, we observed that 53BP1 levels were significantly higher in Ku70-ΔSAP cells compared to WT cells, although the intensity of 53BP1 reached to a similar level between the two cell types by 30 min (Supplementary Fig. S7), in agreement with a less optimal early repair of damage.

### Lack of SAP domain dampened the chromatin association of LIG4 and XLF in human cells

**Figure 8.**
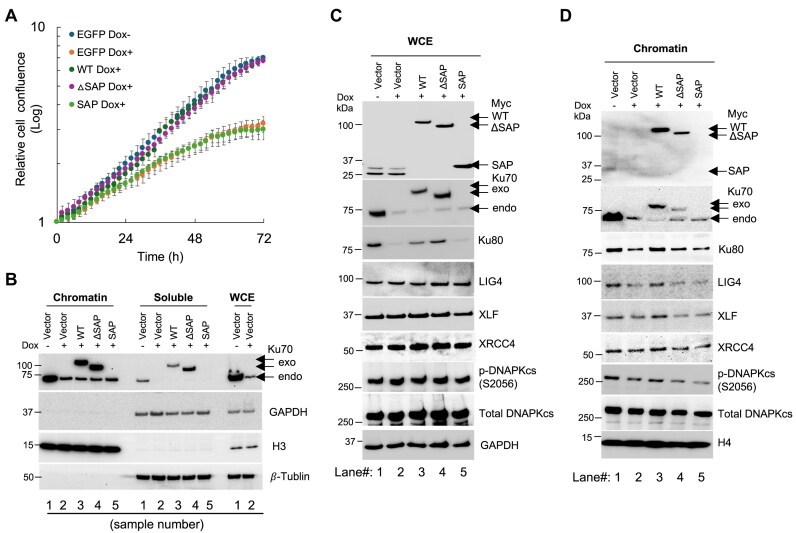
Ku70-ΔSAP reduces recruitment of NHEJ co-factors to chromatin in the H1299 cell line following IR. H1299-ishKu70 cells stably expressing exogenous (exo) Myc-EGFP-tagged Ku70 (WT and mutants) were treated with Dox (Dox+) for 4–7 days to induce the knockdown of endogenous (endo) Ku70, with Dox− serving as a control for comparison. (**A**) Cell growth curves of the indicated cell lines were measured using the IncuCyte live-cell imager. Following Dox pretreatment, the cells were replated to monitor cell growth every 2 h for a period of 72 h. Error bars: SEM. (**B**) The cells were lysed and subjected to a chromatin fractionation assay to validate the effective separation of chromatin and soluble components. GAPDH and β-tublin were used as a reference control for the soluble fractions containing both nuclear and cytoplasmic proteins, while histone H3 served as a positive reference for the chromatin fraction. Whole cell extract from H1299-ishKu70 cells without exogenous Ku70 was loaded to verify the Dox-induced knockdown. The sample numbers are indicated at the bottom of the panels. Western blot analysis of whole cell extracts (**C**) and whole chromatin-associated fraction (**D**) from H1299-ishKu70 cells expressing different Ku70 variants (WT and mutants) at 30 min after exposure to 6.5 Gy of irradiation. The status of Dox treatment is indicated on the top of the panels, and the lane numbers are indicated at the bottom of the panels. The blots were probed with indicated antibodies. WCE: whole cell extracts. The relative intensities of LIG 4 and XLF in chromatin fractions were evaluated in Supplementary Fig. S10. See the “Materials and methods” section for details on radiation exposure.

After observing reduced retention of LIG4 at micro-irradiation-induced DSBs in iMEFs with SAP domain deletion, we performed a co-immunoprecipitation (co-IP) assay. The results showed that the deletion had little effect on Ku70’s ability to interact with LIG4 (data not shown). We then aimed to investigate whether deletion of the Ku70 SAP domain affects LIG4 retention in association with chromatin after radiation exposure. Recognizing the essential role of human Ku70 in cell viability, we used the inducible Ku70 knockdown H1299-ishKu70 cells. After reintroducing various exogenous human Ku70 variants (Supplementary Fig. S2), including Ku70-WT, Ku70-ΔSAP, and Ku70-SAP, into H1299-ishKu70 cells, we monitored the growth rates upon Dox treatments (Fig. 8A). The expressions of these Myc-EGFP-tagged Ku70 variants (Fig. 8B and C) and their nuclear localization were verified in the cells using Myc-EGFP tag (Supplementary Fig. S8). As anticipated, the exogenous expression of Ku70-WT fully restored the proliferation defect induced by endogenous Ku70 depletion following Dox treatment (Fig. 8A). Interestingly, Ku70-ΔSAP showed a growth rate similar to Ku70-WT, indicating that the deletion of the human SAP domain did little to impact cell growth (Fig. 8A), aligning with the normal development observed in *Ku70-ΔSAP* mice (Supplementary Fig. S3). However, the expression of the Ku70 SAP domain alone failed to rescue cell growth retardation (Fig. 8A), underscoring that the SAP domain itself is insufficient to sustain cell viability despite being localized in the nucleus (Supplementary Fig. S8).

After confirming that the Dox treatment (lanes 2–5, Fig. 8B and C) significantly reduced endogenous Ku70 as compared to Dox untreated (lane 1, Fig. 8B and C), we performed protein fractionation to isolate the whole chromatin-associated proteins (S3W) using the procedure outlined in Supplementary Fig. S9. As shown in Fig. 8B, the whole chromatin fraction had little cross-contamination from soluble components such as GAPDH and tubulin, while histone H4 is retained in the chromatin fraction as expected. We then compared the chromatin-associated Ku70 protein variants along with other NHEJ co-factors, among the cells expressing different Ku70 variants with an emphasis on irradiated cells. As shown in Fig. 8D, Dox treatment of cells with empty vector (lane 2, Dox+) was able to reduce the chromatin-associated Ku70 and Ku80, and this was associated with decreased levels of chromatin-associated LIG4 and XLF, compared to the same cells without Ku70 knockdown (lane 1, Dox−). The re-expression of Myc-EGFP tagged WT Ku70 largely restored the chromatin-associated LIG4 and XLF (lane 3, Fig. 8D) to a similar level as the H1299-ishKu70 cells without Dox treatment (lane 1, Fig. 8D). However, the reintroduction of Ku70-ΔSAP and Ku70-SAP (lanes 4 and 5 of Fig. 8D) did not restore the levels of chromatin-bound LIG4 and XLF to the same level as Ku70-WT could (lane 3, Fig. 8D), despite the total protein levels being similar to Ku70-WT cells (lanes 4 and 5, Fig. 8C). After loading normalization with the histone from three independent experiments, the chromatin-associated LIG4 and XLF showed significant reductions in Ku70-ΔSAP cells compared to Ku70-WT cells (Supplementary Fig. S10). However, the quantification did not reveal a significant difference of chromatin-bound XRCC4 and p-DNA-PKcs (S2056) between Ku70-WT and Ku70-ΔSAP cells (data not shown).

The analyses in Fig. 8B and D were conducted using the procedure that separates the soluble and whole chromatin-associated protein (S3W) fraction as illustrated in Supplementary Fig. S9. Considering that many DNA repair factors, including portions of Ku, LIG4, and XLF, may be loosely associated with chromatin without DNA damage exposure, we adapted additional fractionation steps to separate the whole chromatin-associated protein into loosely associated (or mobile) fraction (S3L) and the tightly associated chromatin fraction (S3T) using the procedure in Supplementary Fig. S9 (bottom). Based on Herrmann *et al.* [23], treating nuclear extracts with salty IV150 buffer effectively removes loosely chromatin-associated protein such as HMGB1 [37], while retaining tightly chromatin-associated proteins like histones. Thus, we used IV150 salt buffer to stripe away loosely binding proteins, followed by extracting tightly bound proteins by dissolving the chromatin in 0.2 N HCl (Supplementary Fig. S9). As shown in Supplementary Fig. S11A, a small portion of Myc-EGFP-Ku70-WT and mutants, along with most of HMGB1, were extracted from chromatin upon treatment with 150 mM salt and were found in the S3L fraction. In contrast, the majority of chromatin-associated Ku70 and histone H3 remained resistant to this salt concentration and were recovered in the S3T fraction. Thus, we analyzed the retention of Ku70, LIG4, and XLF in S3T fraction (Supplementary Fig. S11B). Upon radiation exposure, re-expression of WT Myc-EGFP-Ku70 (lane 8 of Supplementary Fig. S11B) restored the chromatin binding of LIG4 and XLF to levels similar to those in control cells without knockdown (lane 6 of Supplementary Fig. S11B, Dox−). Conversely, re-expression of Ku70-ΔSAP or SAP alone (lanes 9 and 10 of Supplementary Fig. S11B) resulted in levels of LIG4 and XLF as comparable to the basal level of empty vector control (lane 7 of Supplementary Fig. S11B, Dox+ with empty vector). Notably, LIG4 and XLF were also associated with chromatin in the absence of irradiation (lane 1 of Supplementary Fig. S11B), albeit at lower levels than in irradiated cells (lane 6 of Supplementary Fig. S11B). These findings reinforce our conclusion that the absence of the SAP domain reduces the chromatin association of LIG4 and XLF following radiation exposure.

Together, our data (Figs 7 and 8, and Supplementary Fig. S11) strongly suggest that the deletion of the SAP domain dampens the chromatin association of LIG4, and XLF to a less extent, offering a novel mechanism for Ku70, especially its SAP domain, to regulate a critical step of NHEJ after formation of the DNA-PK complex.

## Discussion

Despite previous efforts to elucidate the structural characteristics of Ku70-SAP and the biochemical understanding that the Ku70-SAP may bind to RNA or DNA [16–18] and limited cellular analyses with C-terminal deletions containing the SAP domain [24, 25], the specific function of the mammalian Ku70-SAP has not been well documented in the literature. In a recent study [17], the *A. thaliana* Ku70-SAP was found to facilitate the DNA end loading of Ku dimers but it was not required for classical NHEJ and telomere maintenance in the plants. Because the SAP deletion in the *A. thaliana* study retained the nSAP domain [17], in addition to that *A. thaliana* does not have a DNA-PKcs [7] and thus may use a distinct Ku-dependent mechanism to repair DSBs, it is of particular interest for DNA repair and cancer research fields to directly define the function of mammalian Ku70-SAP.

In this study, we investigated the deletion of both the Ku70-specific nSAP and the generically defined cSAP of mouse Ku70 and found that these deletions affected little of the gross animal development and the physiological CSR and V(D)J recombination process. However, they did have effects on animal sensitivity to IR, chromosome aberrations, and cellular sensitivity to radiation exposure or chemotherapy drugs. We further showed that SAP deletion led to a decreased efficiency of the Ku70-ΔSAP in reaching DNA damage sites in cells. Additionally, there was a dampened early retention of LIG4 at the DNA damage sites upon SAP deletion. Our discoveries are significant as they indicate that the SAP domain plays a role in cellular sensitivity to DNA damage. Therefore, targeting the SAP domain for inhibition could potentially enhance cell sensitivity to radiation and other therapeutic agents that induce DSBs, with minimum adverse effects on the physiological processes of animal growth and immunological development. While methylation of Ku70-nSAP domain was previously shown to be critical for Ku70 to translocate to cytoplasm to inhibit apoptosis [22], it is not clear whether this mechanism also contributes to the overall sensitization of Ku70-ΔSAP cells to DNA damage since the deletion of the entire SAP domain may not necessarily exhibit the same functional outcome as the loss of the methylation of SAP domain.

Recently, there have been major breakthroughs in the structural understanding on how the DNA-PK complex and associated dynamic proteins execute the ligation of two dsDNA ends [8, 12]. The consensus model is that following the encircling of dsDNA by the Ku dimer and the recruitment of DNA-PKcs to protect the DNA end, the Ku dimer shifts inward from the break end to stabilize the DNA [38–40]. However, there are alternative configurations of protein complex after the initial DNA-PK complex is established. For examples, this complex can recruit LIG4/XLF/XRCC4 and other proteins to form either domain-swap or XLF-mediated conformations, each with distinct activities tailored toward different types of DNA ends [8]. Therefore, it is plausible that distinct evolutionary steps have been developed to address various DNA strand breaks, and a defect in one specialized conformation may not necessarily impact the function of another, potentially resulting in different repair outcomes based on the types of DSBs. Our results suggest that SAP domain is essential for a conformation that favors the repair of radiation- and chemotherapy agent-induced DSB, but less critical for the ones to perform CSR and V(D)J recombination. This highlights the intricate and specialized roles that different configurations of the protein complex play in DNA repair processes, depending on the nature of the DNA damage encountered.

The next question of why and how Ku70-SAP is required for repairing of externally applied DSBs but with little impact on CSR and V(D)J recombination deserves a deeper exploration. While the specific mechanism remains unclear, two alternative but nonexclusive possibilities may explain this observation. One potential contributing factor is the difference of chemical structures at the broken DNA ends. A distinction between DSBs generated during CSR and the V(D)J and those induced by exogenous insults is the chemical nature of the break ends. The CSR and V(D)J ends are processed by cleavage of the DNA with enzymes that can directly result in the ligation compatible ends. In contrast, the IR- and chemotherapy-induced DSBs are likely to have un-uniformed chemical structure at the ends, which would require extra-end processing steps before they become compatible with later steps. This may require an extra regulatory step that is fulfilled by the SAP domain. An alternative scenario is that Ku70-SAP is required for optimal repair of DNA damage. Without the SAP domain, Ku70 can effectively manage the physiological level of DSBs that are well regulated. However, the SAP-less cells struggle to adequately cope with the sudden and excessive DSBs generated by radiation or chemotherapy drugs, resulting a sensitization to these. Clearly, the specific mechanism by which SAP deletion renders cells sensitive to externally applied DSBs, but has little impact on CSR and V(D)J related to strand breaks, would need further investigation.

Delving into a more detailed analysis, the present study has provided new support for a conceptual refinement of the Ku70-SAP domain that holds functional significance. As illustrated in Supplementary Fig. S1, the commonly referred Ku70-SAP is composed of two distinct sub-domains. It is only the cSAP that aligns with the originally defined SAP motif of ∼35 aa, but the 14 aa nSAP forms a new helix that appears to have evolved specifically for Ku70, although the nSAP and cSAP together form a globular structure. Interestingly, among all possible positions of the Ku70-SAP, it is the nSAP helical domain that serves as the interaction interface between the Ku70-SAP and the main body of the Ku dimer [9, 10, 15]. Thus, we speculate that the Ku70-nSAP likely evolved to mediate certain Ku70 specific function via its interaction with the Ku dimer and others, while the cSAP fulfills the more generic functions commonly associated with SAP domains. This concept emphasizes the complex interaction dynamics between the distinct sub-domains of the Ku70-SAP, underscoring their specific functions in facilitating precise protein interactions within the DNA repair complex.

The current results further suggest that one way for the SAP domain to achieve these functions is via optimal retention of early phase LIG4 at the DNA damage sites, and also likely its associated partners, which could impact its ligase activity. This regulatory function is particularly relevant during the transition of LIG4 from a nonenzymatic role in tethering DNA ends within the long-range synapsis complex to a state ready for ligation within the short-range synapsis complex. This transition likely necessitates a reconfiguration of LIG4 to enable its flexible ligase domain to access the ends that are suitable for ligation. Coincidentally, the nSAP helix was shown to dock around the α/β-domain of Ku80 [9, 10] or the Ku aperture area [15], which overlap with the Ku80 core region and the Ku70 C-helical ARM that bind to the LIG4 [6], while the cSAP has not been implicated in any of these interactions.

Early phase LIG4 retention was dampened at micro-irradiated stripes as well as in the chromatin-associated form in irradiated cells at an intermediate time frame after DNA damage (Figs 7 and 8D, and Supplementary Fig. S11). Our observation may indicate a role of the SAP domain in the stability of complex through the retention of LIG4, a prediction requiring additional experimental testing. LIG4 has at least two roles in the NHEJ. First, two LIG4, two XRCC4, and an XLF homodimer form the long-range synapsis complex to tether two DNA ends. This function does not need the ligase activity [31] but is still critical for NHEJ. When transitioning to the short-range complex, the flexible ligase domain of LIG4 must be reconfigured to access the synapsed DNA ends, allowing each LIG4 molecule to ligate one strand and facilitate the rejoining of the DSB ends. Interestingly, this LIG4 enzymatic activity appears be dispensable for NHEJ, likely due to LIG3 or other yet-to-be-identified compensating ligase activity [41]. Because SAP loss impaired LIG4 retention, which would also call for an alternative ligase to rejoin the break as proposed by Goff *et al.* [41], our results hint at a potential synthetical lethal relationship between the SAP domain and the putative LIG4 backup ligase during the NHEJ. Furthermore, it is noteworthy that the nSAP binding interfaces on the Ku dimer appear to overlap with some of the binding surface of LIG4. Thus, it is possible that the SAP domain may coordinate with LIG4 to reconfigure the flexible ligase domain to promote end ligation. This prediction is consistent with the structural model where the nonenzymatic LIG4 is crucial to form and maintain the long-range and short-range synapsis complexes, but the enzymatic domain of LIG4 is reconfigured to localize and ligate one strand of the DNA, which seems compensable by other ligase(s) when the enzymatic activity of LIG4 is inactivated. We also observed an intriguing phenomenon of widened γHA2X stripes along the micro-irradiated regions. The exact implications of this broadened pattern remain to be explored. However, γH2AX plays a vital role in the response to DSBs. It aids in reorganizing chromatin and retaining essential factors at the sites of DSBs [42]. In *Ku70-ΔSAP* cells, one possible cause for the diffuse γH2AX pattern is the persistent DNA damage signals resulting from delayed DNA repair, which may be due to sub-optimal recruitment of Ku70 and retention of LIG4. This ongoing DNA damage signaling could further lead to changes in chromatin structure.

Overall, the present findings are exciting as they have demonstrated that SAP deletion confers a sensitivity to DNA damage from irradiation and DSB-inducing chemotherapy agents, while having little impact on physiological DSB repair. This suggests that specific disruption of SAP function could be a promising strategy to enhance cell sensitivity to therapeutic DNA damage, while devoid of adverse effects on the physiological activity of Ku70 in animal development and immunological response. Our functional and cell biology analyses also raise interesting questions for biochemical and structural refinement of the NHEJ process, as well as open new opportunities to manipulate the NHEJ process for therapeutic development.

## Supplementary Material

gkaf499_Supplemental_File

## Data Availability

The data underlying this study will be deposited to public database per NIH policy and can be made available to readers.
