# Supplementary material for: The mammalian Ku70 C-terminus SAP domain is required to repair DNA damage
Source: Nucleic Acids Res. 2025 Jun 11;53(11):gkaf499. doi: 10.1093/nar/gkaf499 (PMC12153343; doi:10.1093/nar/gkaf499)
Supplement: gkaf499_Supplemental_File [file gkaf499_supplemental_file.pdf]

Supplemental Information

(Wang et al. The Mammalian Ku70 C-terminus SAP Domain Is Required to Repair DNA Damage)

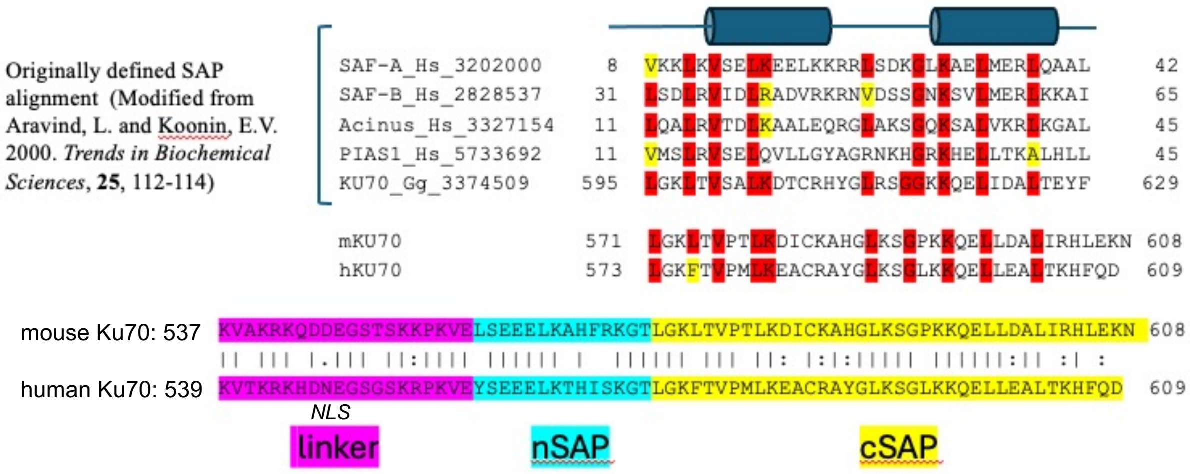

Figure S1. Distinction between the canonical SAP (cSAP) and the nSAP domains of Ku70. The cSAP of human and mouse Ku70 can be aligned with the originally defined SAP, but the nSAP appears to be Ku70-specific.

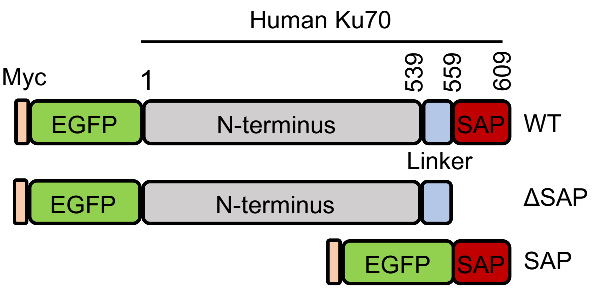

Figure S2. Schematic representation of Myc-EGFP tagged full length Ku70 (WT) and two truncated mutants (ΔSAP and SAP).

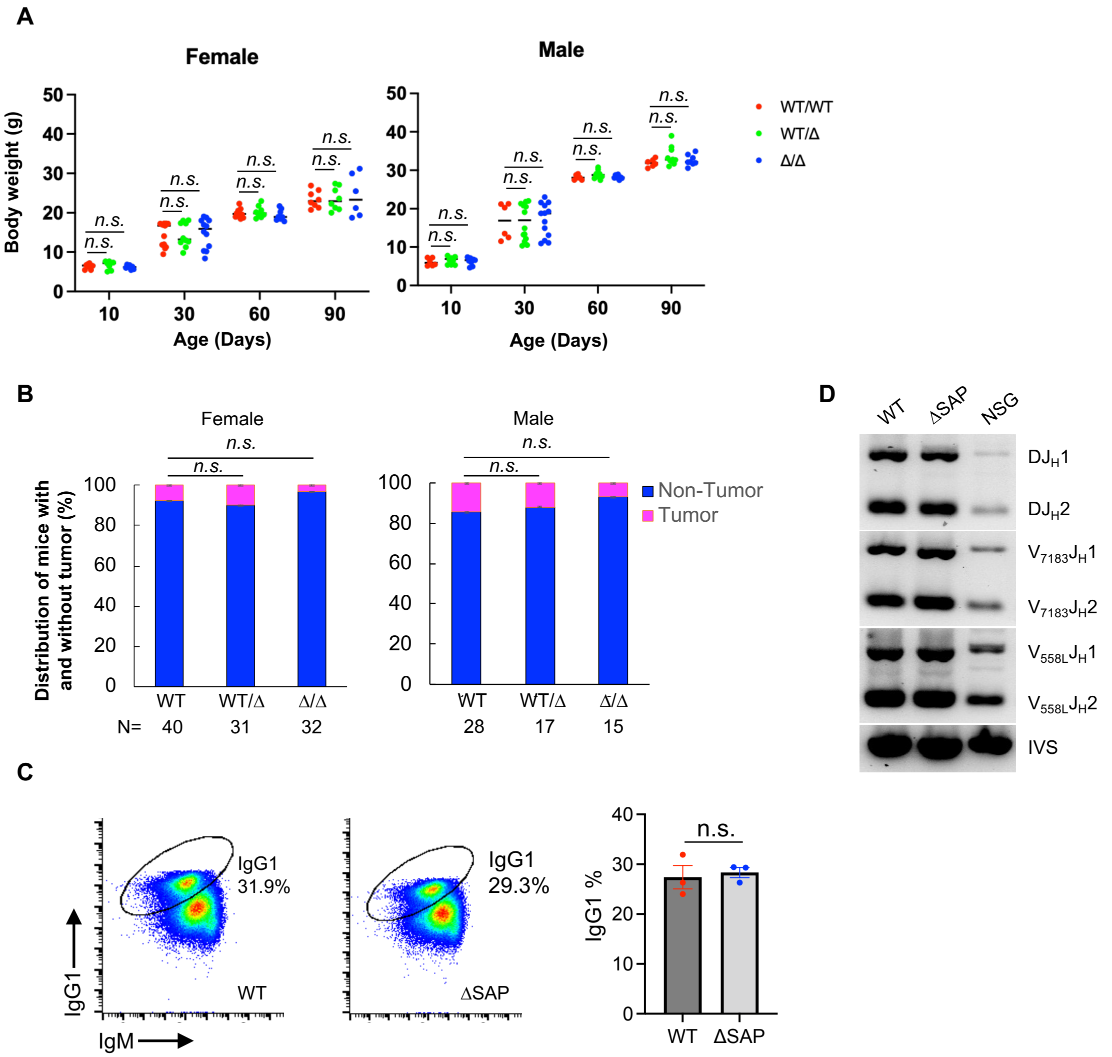

Figure S3. Absence of significant spontaneous developmental defects and tumor burden in ΔSAP mice. (A) The body weight of WT and ΔSAP mice was recorded over the first 3 months after birth. (B) The distribution of mice with or without the tumors was assessed in both WT and ΔSAP mice. Mice were sacrificed at 600 days to evaluate the presence of spontaneous tumors or other abnormalities. (C) Immunoglobulin class switching in stimulated B cells was measured by the frequency of IgG1. The left two panels show representative flow cytometric profiles of IgG1 conversion from IgM in WT and ΔSAP mice. The right panel summarizes the frequency of IgG1 in IgM^+ cells based on three experiments. P values were calculated using a two-tailed Student's t-test. *n.s.* denotes non-significant. (D) PCR products of D_H to J_H, and V_{7183}, V_{558L} to DJ_H gene segments recombination in KU70-ΔSAP mice. Genomic DNA was extracted from BM cells of indicated mouse lines, 100 ng DNA was used for PCR each line. Abbreviations: NSG mouse: NOD Scid gamma mouse; IVS: Intervening sequence (nonrecombining segment of the Ig locus between J_H and C_H1). The same primers reported by Ouyang et al were used (Ouyang 1997, J Exp Med, 186:921, PMC2199057).

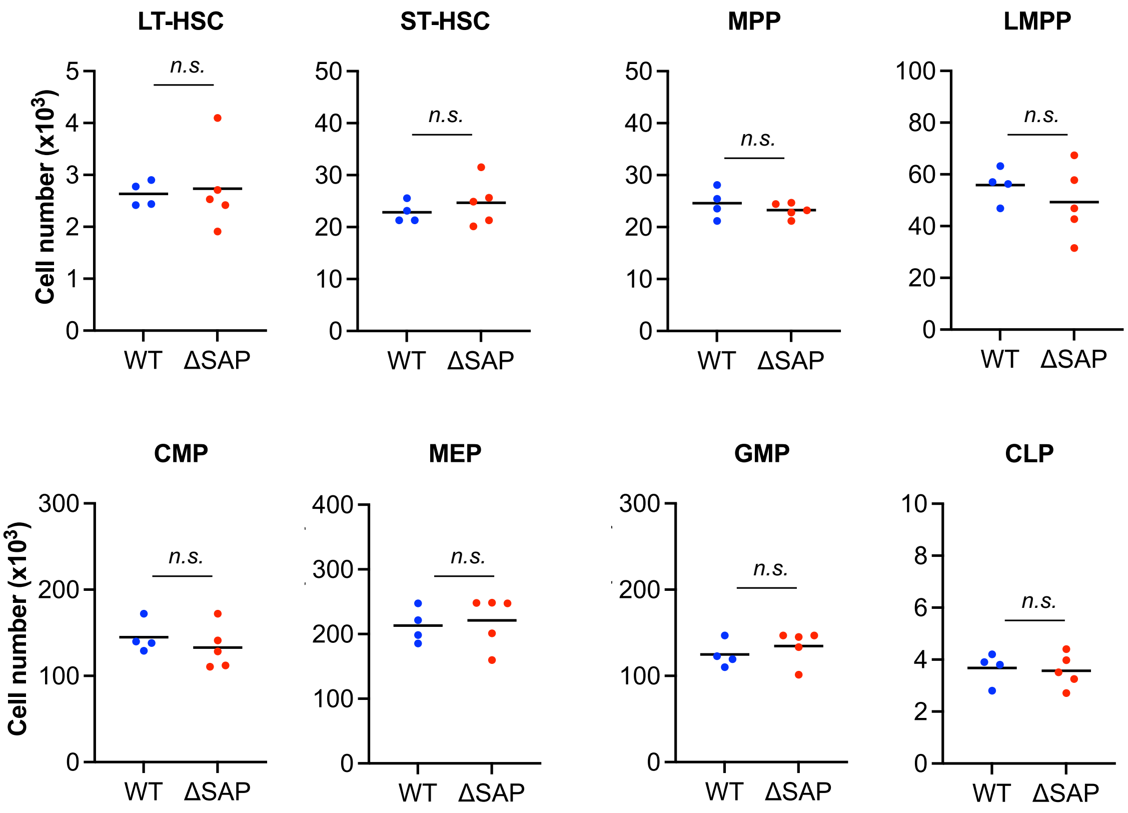

Figure S4. Analysis of hematopoietic stem cells (HSCs) and progenitor cells in the bone marrow (BM) of No IR group. The counts of HSCs and progenitor cells in the BM of eight-week-old male mice that were not exposed to TBI. The BM cells were collected, and Lineage negative (Lin^-) cells were isolated. The flow cytometry gating strategy identified various cell populations as indicated in Figure 3A. The total numbers of each cell populations are presented as means (n = 4-5 mice/group). *n.s.* denotes non-significant by unpaired two-tailed Student's t test.

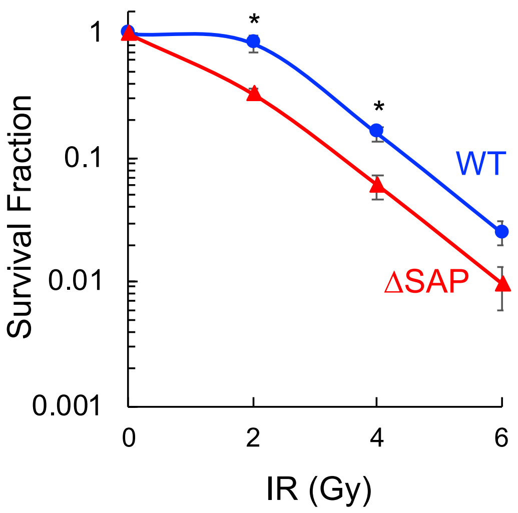

Figure S5. Radiation sensitivities of H1299 cells. H1299-ishKu70 cells stably expressing Myc-Ku70-WT and Myc-Ku70-ΔSAP were pre-treated with Dox for 7 days to induce knock-down of endogenous Ku70 before colony formation assays. Error bars are the standard errors of the means (SEMs) of 6 dishes. P values were calculated based on two-tailed student t test. *$p \leq 0.05$, *n.s.*, non-significant.

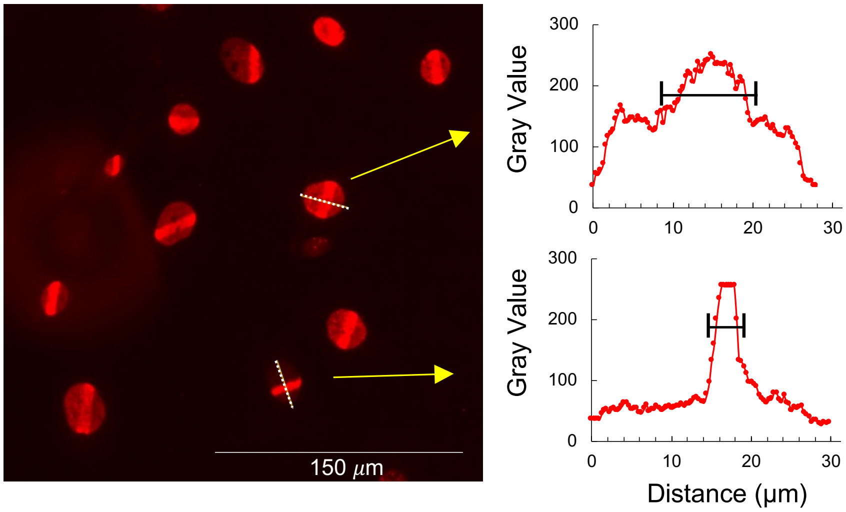

Figure S6. Measurement of γH2AX stripe width in individual cells was performed by drawing a perpendicular line across the irradiated stripe in each cell. The signal intensity was then plotted along the line, and the width of the signal peak was measured for each cell. The image shown illustrates representative cells with both sharp and diffuse γH2AX stripes following micro-irradiation in Ku70-ΔSAP cells.

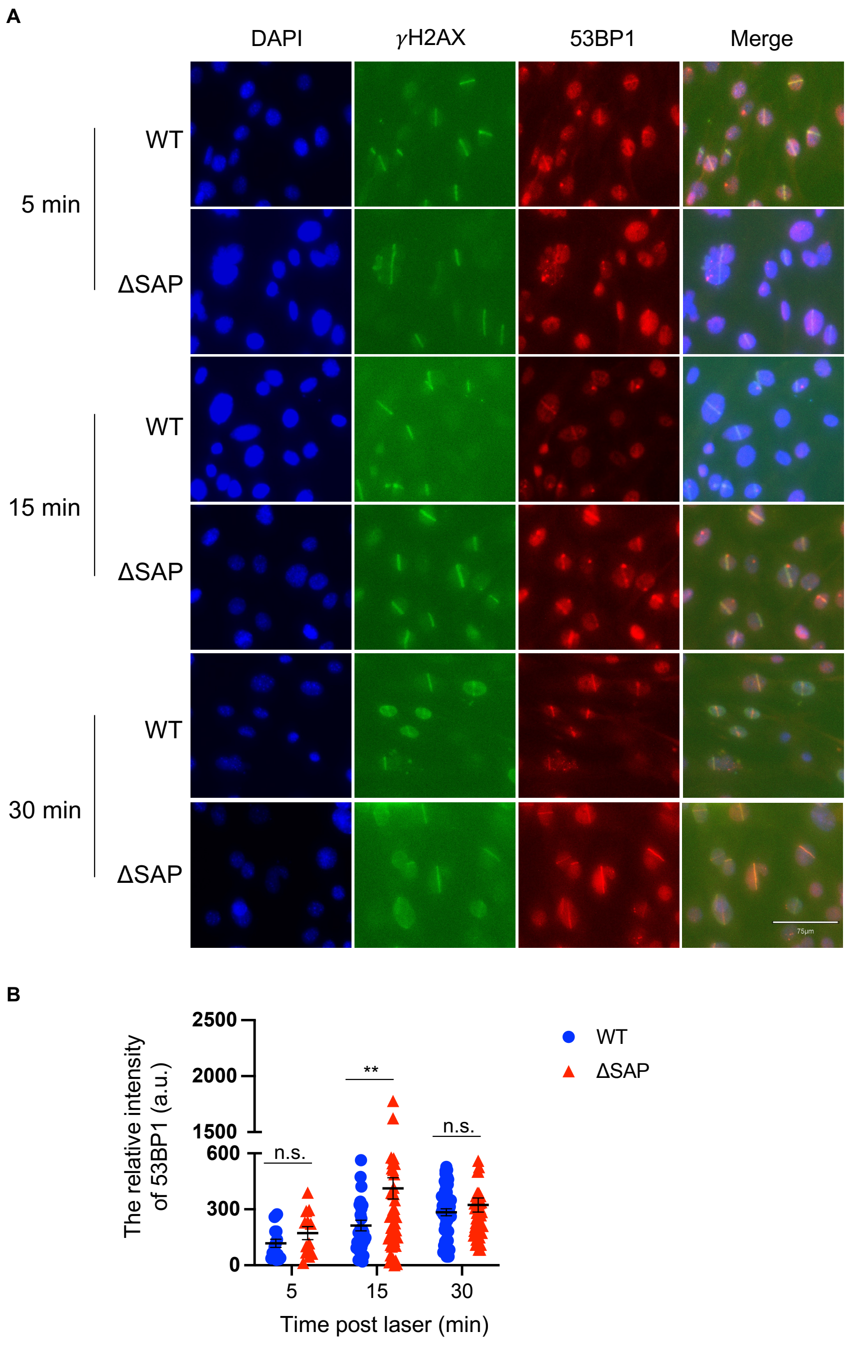

Figure S7. 53BP1 recruitment to micro-irradiation induced DNA damage sites in iMEFs. (A) Representative IF staining images of γH2AX (green) and 53BP1 (red) post micro-irradiation at the indicated time points. Cell nuclei were visualized with DAPI (blue). Scale bars, 75 μm. (B) The relative fluorescence intensity of 53BP1 in WT and ΔSAP MEFs at the irradiated sites were quantified at the indicated time points post micro-irradiation. Error bars represent the standard errors of the means (SEMs). P values were calculated using a two-tailed Student's t-test. $^{**}p \leq 0.01$, *n.s.*, non-significant.

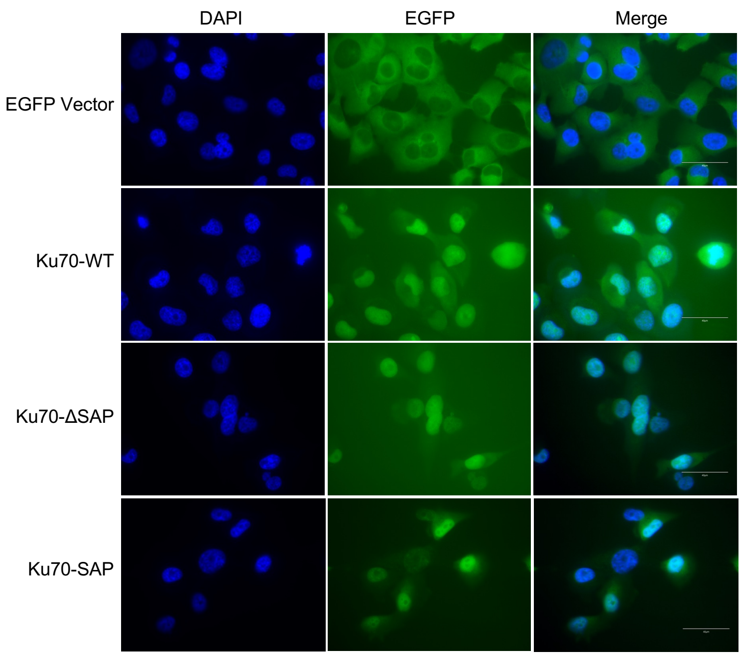

Figure S8. Intra-cellular localization of Myc-EGFP tagged Ku70 (WT and mutants) in H1299 cells. Scale bars, 40 µm.

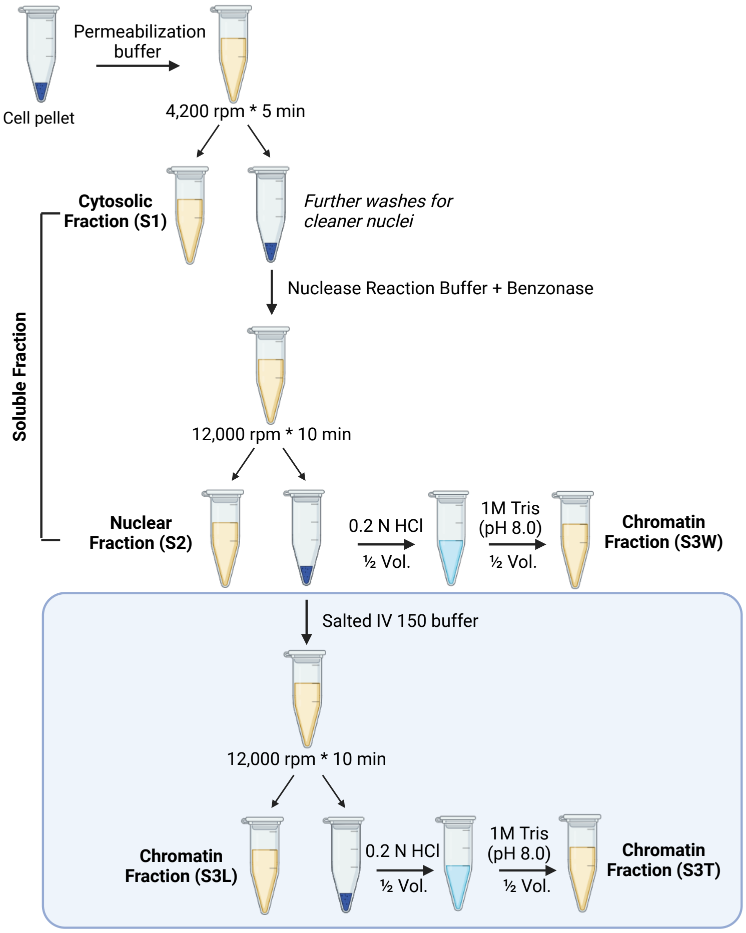

Figure S9. Scheme of the protein fractionation procedure. S3W (whole) includes all proteins associated with chromatin after nuclease treatment, which can be further fractionated into S3L (Loose) for loosely associated chromatin proteins and S3T (Tight) for tightly bound chromatin proteins by treatment of 150 mM salt buffer. Vol indicates volume.

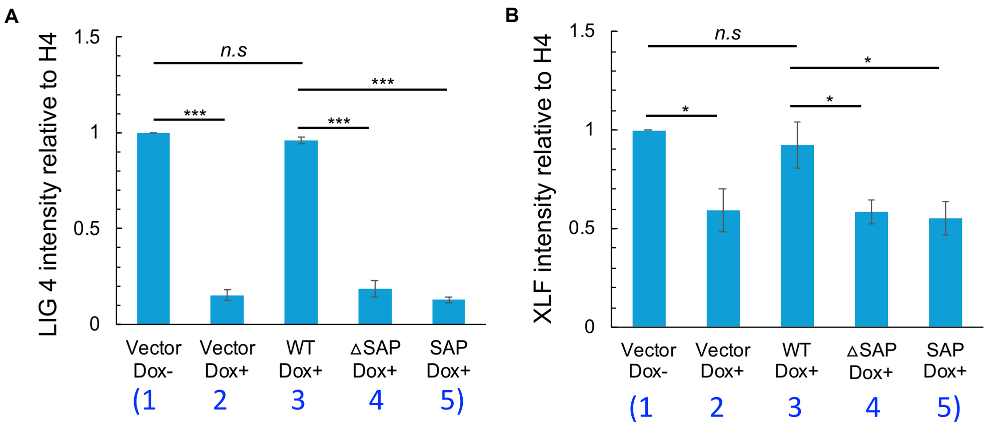

Figure S10. Quantification of chromatin-bound proteins in H1299-ishKu70 cells. The band intensities of LIG4 (A) and XLF (B) from Figure 8D, along with 2 more repeats, were quantified using ImageJ software and normalized to histone (a loading control for chromatin protein) of the same lanes. Shown are the averages of the ratios presented as arbitrary unit normalized to lane-1 (Vector Dox- control group), which was set to 1.00. Results represent the average of three independent experiments. Error bars, SEM. Two-tailed *t-test* was used for calculating *p* values. ***$p \leq 0.001$, *n.s.*, non-significant.

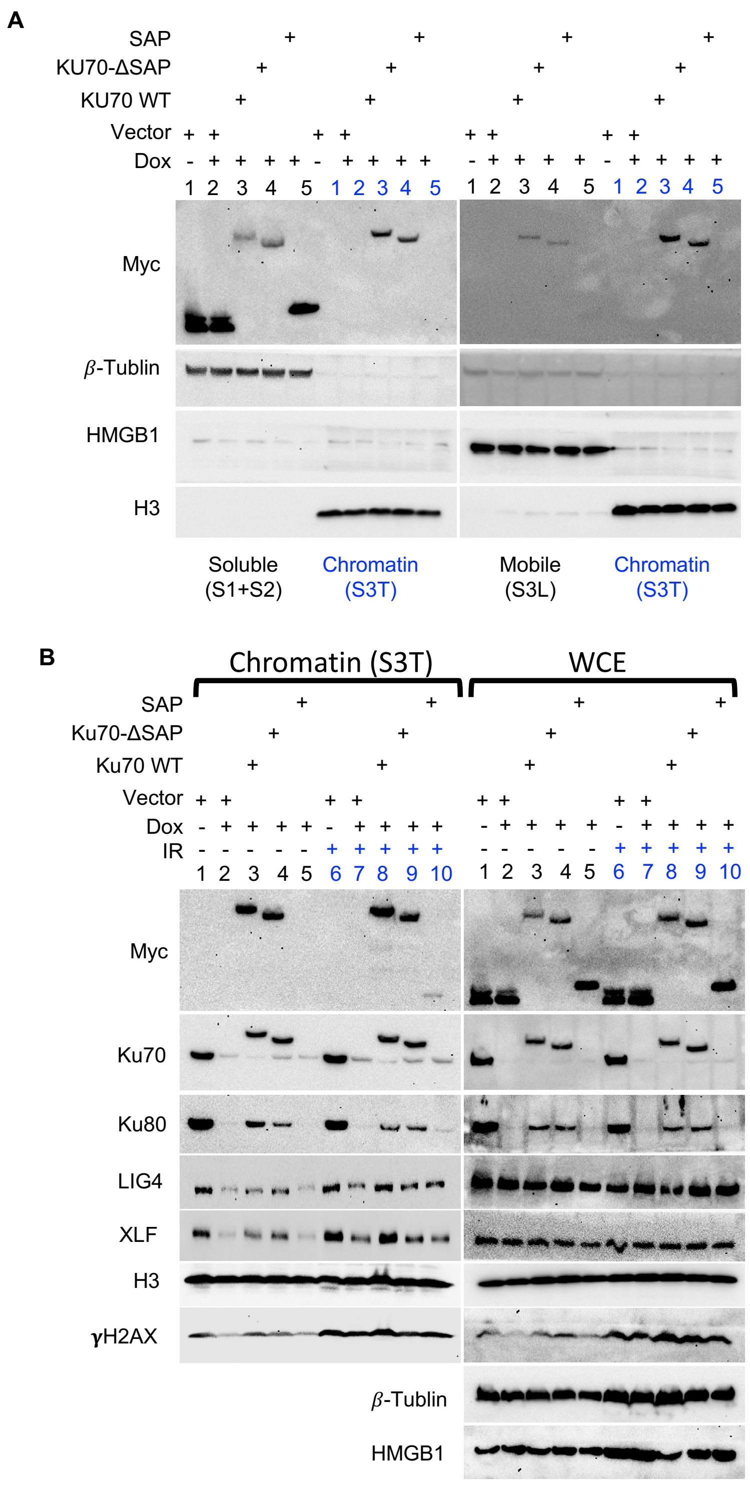

Figure S11. Lack of Ku70 SAP reduces tightly chromatin-association of LIG4 and XLF after irradiation. The same panel of H1299-ishKu70 cells expressing Myc-EGFP-tagged Ku70 (WT and mutants as illustrated in Figure S2 and used in Figure 8), was irradiated under identical conditions and subjected to fractionation analysis. (A) Cells were lysed and separated into three fractions: non-chromatin proteins (soluble, S1+S2), loosely associated chromatin proteins (mobile, S3L), and tightly associated chromatin proteins (S3T), using the procedure outlined in Figure S9. Immunoblots showing selected markers for each fraction are displayed. β-Tubulin served as a reference for the soluble fraction, histone H3 as a loading control for the tightly bound chromatin fraction, and HMGB1 as a marker for loosely associated chromatin proteins extractable with 150 mM salt buffer. The majority of Myc-EGFP-Ku70 was found in the S3T fraction, which was used to analyze the chromatin retention of selected NHEJ factors. (B) Western blot analysis of the tightly chromatin-bound fraction (S3T, left) and whole cell extracts (WCE, right) from H1299-ishKu70 cells was performed 30 minutes after treatment with 6.5 Gy IR or without.

Table S1. Complete blood count from Ku70-WT and Ku70-ΔSAP Mice. Blood samples were drawn from the submandibular vein of 180-day old mice (N=3-5), collected in BD vacutainer EDTA tubes (#367844) and analyzed with the HESKA-HT5 veterinary hematology analyzer. No significant statistical differences were observed in the results. Abbreviations: WBC, white blood cells; Neu, neutrophils; Lym, lymphocytes; Mon, monocytes; Eos, eosinophils; Bas, basophills; RBC, red blood cells; HGB, hemoglobin; HCT, hematocrit; MCV, mean corpuscular volume; PLT, platelets; MPV, mean platelet volume.

Parameter	WT	ΔSAP
WBC ($10^3/\mu L$)	10.20 ± 0.21	9.34 ± 1.32
Neu (%)	11.45 ± 0.49	13.98 ± 1.40
Lym (%)	86.38 ± 0.05	82.73 ± 1.33
Mon (%)	1.28 ± 0.41	1.57 ± 0.12
Eos (%)	0.51 ± 0.11	1.03 ± 0.18
Bas (%)	0.37 ± 0.03	0.68 ± 0.02
RBC ($10^6/\mu L$)	9.35 ± 0.56	10.14 ± 0.07
HGB (g/dL)	14.26 ± 0.91	14.90 ± 0.06
HCT (%)	42.16 ± 2.43	45.13 ± 0.22
MCV (fL)	45.10 ± 0.38	44.45 ± 0.09
PLT ($10^3/\mu L$)	1207.67 ± 153.59	1419.67 ± 170.57
MPV (fL)	5.47 ± 0.06	5.43 ± 0.03

Table S2. Summary of metaphase chromosomal abnormalities in Ku70-WT and Ku70-ΔSAP mouse splenic B cells, with or without IR treatment.

Aberration Types	0 Gy		2 Gy	
	WT	ΔSAP	WT	ΔSAP
Premature Centromeric Separation (PCS)	2	9	24	54
Dicentric Chromosomes (DC)	1	2	6	15
Chromatid Break (CB)	5	19	28	60
Fragment (F)	2	4	9	53
Acentric Chromosome (AC)	1	6	5	15
Translocation (T)	0	3	0	0
Sister Chromatid Fusion (SCF)	0	9	3	9
End To End Fusion (ETE)	0	0	2	7
Quadriradial (QUA)	0	0	0	3
Triradial (TRI)	1	1	2	3
Premature Chromatid Separation (PCH)	1	7	7	8
Isochromosome (ISO)	0	6	32	45
Total Number of Aberrations	13	66	118	272
Total Spreads	101	305	140	245
Average Aberrations per Metaphase Spread	0.12	0.22	0.84	1.11
